# Bridging the gap: molecular mechanisms, regional activity and connectivity in headache disorders

**DOI:** 10.1093/brain/awaf361

**Published:** 2025-09-25

**Authors:** Linda Kollenburg, Erkan Kurt, Wim Mulleners, Hisse Arnts, Christopher Louis Robinson, Janneke Poelen, Kaare Meier, Moises Dominguez, Sait Ashina, Kris Vissers

**Affiliations:** Department of Neurosurgery, Radboud University Medical Center, Nijmegen 6525 GA, The Netherlands; Department of Neurosurgery, Radboud University Medical Center, Nijmegen 6525 GA, The Netherlands; The Migraine Clinic, Amsterdam 1083 HA, The Netherlands; Department of Neurosurgery, Radboud University Medical Center, Nijmegen 6525 GA, The Netherlands; Department of Anesthesiology, Perioperative, and Pain Medicine, Harvard Medical School, Brigham and Women’s Hospital, Boston, MA 02115, USA; Department of Neurology, Canisius Wilhelmina Hospital, Nijmegen 6532 SZ, The Netherlands; Department of Neurosurgery, Aarhus University Hospital, Aarhus N 8200, Denmark; Department of Anesthesiology, Aarhus University Hospital, Aarhus N 8200, Denmark; Department of Neurology, New York Presbyterian Hospital, Weill Cornell Medical College, New York, NY 10065, USA; Beth Israel Deaconess Medical Center, Department of Neurology, Harvard Medical School, Boston, MA 02215, USA; Department of Anesthesiology, Pain and Palliative Care, Radboud University Medical Center, Nijmegen 6525 GA, The Netherlands

**Keywords:** connectivity, molecular mechanisms, migraine, cluster headache, hemicrania, occipital neuralgia

## Abstract

Chronic headache disorders have a tremendous impact on psychosocial functioning. Despite the availability of various treatment options, suboptimal management remains present in a subset of patients, leading to persistent suffering. Molecular mechanisms, regional activity patterns and connectivity pathways are crucial for understanding the pathophysiology, serving as a foundation for developing novel treatments, refining existing therapies, and ultimately optimizing the management of headache disorders. Nevertheless, articles combining fundamental and clinical aspects of the pathophysiology and treatment of headache disorders remain limited. The current literature review provides a thorough overview of the molecular mechanisms, regional activity patterns and connectivity pathways involved in migraine, cluster headache (CH), paroxysmal hemicrania (PH), hemicrania continua (HC) and occipital neuralgia (ON), thereby bridging the gap between different fields of expertise.

In this scoping review, literature on molecular mechanisms, regional activity and connectivity pathways for migraine, CH, PH, HC and ON has been collected from the PubMed, MEDLINE and EMBASE databases. Reports were also manually searched using the search function in Google Scholar, as well as reviews or references cited within the articles.

In total, 130 and 97 articles, published between 1976 and 2024, are included in the analysis of the molecular mechanism and regional activity patterns/connectivity pathways, respectively. Molecular data show that the trigeminal nucleus caudalis is a central structure in headache pathology, comprising various neuropeptides and neurochemicals, including vasoactive intestinal peptide, glutamate, substance P and serotonin, and connecting the pathophysiology of these headache disorders. Sensitization of higher cortical brain areas, neuroinflammation within the trigeminal system and vasodilatation of cranial vessels seem to contribute to headache pain. Headache disorders are also associated with atypical regional activity patterns and connectivity pathways in pain processing areas, as well as the default mode network, salience network, and sensorimotor network. These abnormalities help explain the mechanisms underlying overall headache-related symptoms and additional manifestations unique to each headache disorder, including cortical spreading depression in migraine, rhythmicity of attacks in CH and autonomic symptoms in CH, PH and HC.

The article fosters a deeper understanding of the molecular mechanisms, neuronal pathways and clinical symptoms involved in headache pathology across different fields of expertise. By bridging these perspectives, it provides essential insights for developing innovative treatment strategies and enhancing existing therapeutic options.

## Teaser

Chronic headache disorders often respond poorly to treatment. Kollenburg *et al.* review the molecular mechanisms and regional activity patterns underlying conditions including migraine, cluster headache, and paroxysmal hemicrania, integrating basic and clinical perspectives to guide the development of more effective therapies.

## Introduction

Headache disorders are a global burden, affecting almost 50% of the world's population.^1^ In particular, chronic headache disorders are associated with reduced social and physical functioning. They are often accompanied by psychiatric comorbidities like depression, anxiety, and post-traumatic stress disorder, consequently leading to a tremendous impact on patients’ quality of life.^2-4^ Certain chronic headache disorders are even ranked in the World Health Organization's top 10 conditions causing worldwide disability, thus highlighting their significant impact on global health.^5,6^ Migraine, cluster headache (CH), paroxysmal hemicrania (PH), hemicrania continua (HC) and occipital neuralgia (ON) are all well-known headache disorders with different manifestations varying from unilateral to bilateral pain in the frontal, temporal, orbital or nuchal-occipital region, sometimes accompanied by other features like nausea, vomiting, photophobia and unilateral cranial autonomic symptoms (e.g. nasal congestion, conjunctival injection, ptosis, miosis, lacrimation and aural fullness).^6-8^ Over the past 30 years, various treatment modalities have been developed for headache disorders, ranging from non-invasive options such as drugs and non-invasive vagus nerve stimulation (nVNS) to minimally invasive options like botulinum toxin injectables and more invasive neurosurgical approaches like occipital nerve stimulation (ONS) and deep brain stimulation (DBS).^9-11^ Despite these options, in some individuals, headache treatment can be limited by insufficient therapy responses, resulting in a number needed to treat (NNT) of 3–10.^12-15^ To improve the management of these patients, understanding both the fundamental and clinical aspects of the pathophysiology and treatment of headache disorders is crucial, as this may lead to innovative insights into existing and future treatment modalities.^16^ Despite the availability of fundamental and clinical data on this topic, reports combining both perspectives remain scarce. Furthermore, articles covering fundamental details are often considered highly complex, with a limited clinical perspective, which consequently hampers their effectiveness in translational research and in reaching different fields of expertise.

The current literature review aims to provide a clear overview and clinical insights into the molecular mechanisms, regional activity patterns and connectivity pathways involved in migraine, CH, PH, HC and ON. Primary headache disorders (e.g. migraine, CH, PH and HC) are selected for this review as these not only impose a significant socioeconomic burden but are also linked to suboptimal treatment outcomes,^17-20^ underscoring the urgent need for further investigation into their underlying mechanisms. With regard to ON, although its occurrence is relatively rare, it is believed to share overlapping pathophysiological features with primary headache disorders such as migraine.^21^ This overlap, combined with the current challenges in effectively managing ON, underscores the potential for therapeutic improvement and served as a key rationale for its inclusion in the present review, highlighting the need for further research into its underlying pathophysiology. This literature review pioneers the bridging of gaps, not only between molecular mechanisms, regional activity patterns, and connectivity pathways, but also between fundamental data, translational research, and different fields of expertise, as all data are analysed and interpreted from a clinical perspective. To the best of our knowledge, this literature review is the first to provide a thorough written and visual overview, encompassing all the key aspects of the pathophysiology of the headache disorders under investigation.

## Methods

**Figure 1:**
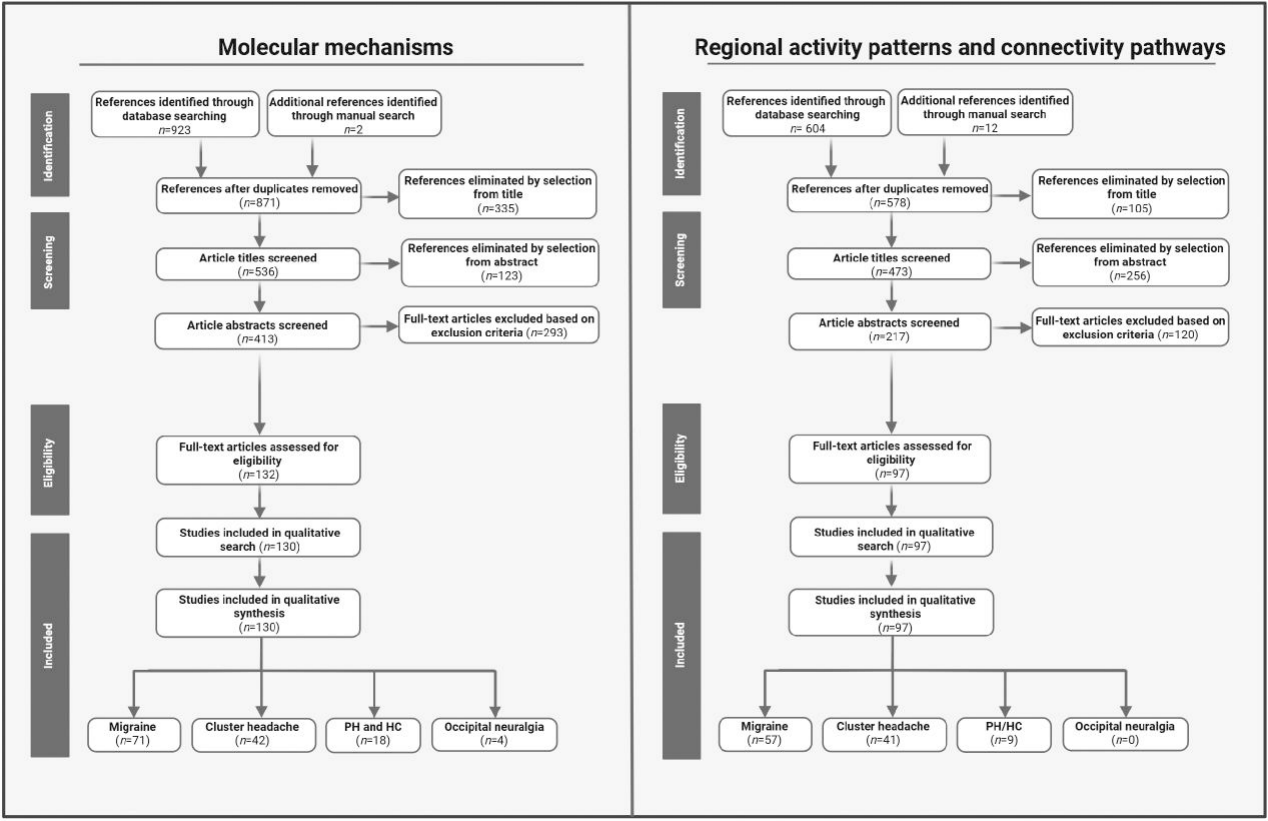
**Literature search.** Overview of literature search performed for molecular mechanisms, regional activity patterns and connectivity pathways in migraine, cluster headache, paroxysmal hemicrania/hemicrania continua and occipital neuralgia. HC = hemicrania continua; PH = paroxysmal hemicrania. *Please note that the column ‘studies included in qualitative synthesis’ reflects the total number of articles analysed within this literature review, with some being listed in more than one headache category in case multiple disorders were covered in the same article. Created in BioRender. Kollenburg, L. (2025) https://BioRender.com/xnrb3zv.

The goal of this scoping review is to describe the molecular mechanisms and neuronal networks involved in the pathophysiology of migraine, CH, PH, HC and ON. To assess these outcomes, a literature search was performed using the PubMed, MEDLINE and EMBASE databases. For the current analysis, two independent searches were performed for the headache disorders: (i) molecular mechanisms; and (ii) regional activity patterns and connectivity pathways (Fig. 1). With regard to the molecular mechanisms, a literature search was performed using the following string: ((‘migraine’ OR ‘cluster headache’ OR ‘occipital neuralgia’ OR ‘hemicrania’) AND (‘molecular’ OR ‘molecular pathways’ OR ‘molecules’ OR ‘pathways’, OR ‘pathology’)). For the search on regional activity patterns connectivity pathways, the following string was used: ((‘migraine’ OR ‘cluster headache’ OR ‘occipital neuralgia’ OR ‘hemicrania’) AND, (‘regional activity’ OR ‘activity’ OR ‘connectivity’ OR ‘functional connectivity’ OR ‘pathogenesis’ OR ‘pathophysiology’ OR ‘pathology’ OR ‘resting state’ OR ‘functional magnetic resonance imaging’ OR ‘imaging’ OR ‘connections’ OR ‘connectivity pathways’ OR ‘pathways’)). Studies were included in this review if they met the following criteria: published in the English language, classified as original research articles or relevant review articles, and investigating molecular pathways, connectivity pathways or regional activity patterns as either primary or secondary outcomes in subjects with migraine, CH, PH, HC and/or ON. Studies were excluded if they were published in languages other than English, involved study populations consisting of species other than humans, non-human primates or rodents, investigated headache disorders other than CH, PH, HC and/or ON, or provided insufficient detail regarding molecular pathways, connectivity pathways, or regional activity patterns. Superseded theories on headache pathology were also excluded from this review. No exclusions were made based on age (studies including paediatric, adult and elderly populations were considered), gender or publication date, as applying such restrictions could lead to the omission of relevant articles and limit the comprehensiveness of the review.

Additionally, reports were also manually searched using the search function in Google Scholar, as well as reviews or references cited within the articles. The current analysis did not include articles that were solely used to find additional studies. All results in each study were sought to be compatible with each outcome domain, including molecular mechanisms, regional activity patterns, and connectivity pathways. All title and abstract records were analysed by two independent reviewers (L.K. and E.K.), and each report retrieved was also analysed by two independent reviewers (L.K. and E.K.). Each study analysed within this literature search was viewed by two independent researchers (L.K. and E.K.) to ensure consensus and the quality of the articles included. In the case of disagreement, additional reviewers (S.A., C.R., W.M. and M.D.) were consulted. Two reviewers collected data from each report (L.K. and E.K.).

## Results

In total, 130 and 97 articles, published between 1976 and 2024, were included in the analysis of the molecular mechanism and regional activity patterns/connectivity pathways, respectively (Fig. 1).

### Molecular mechanisms

**Figure 2:**
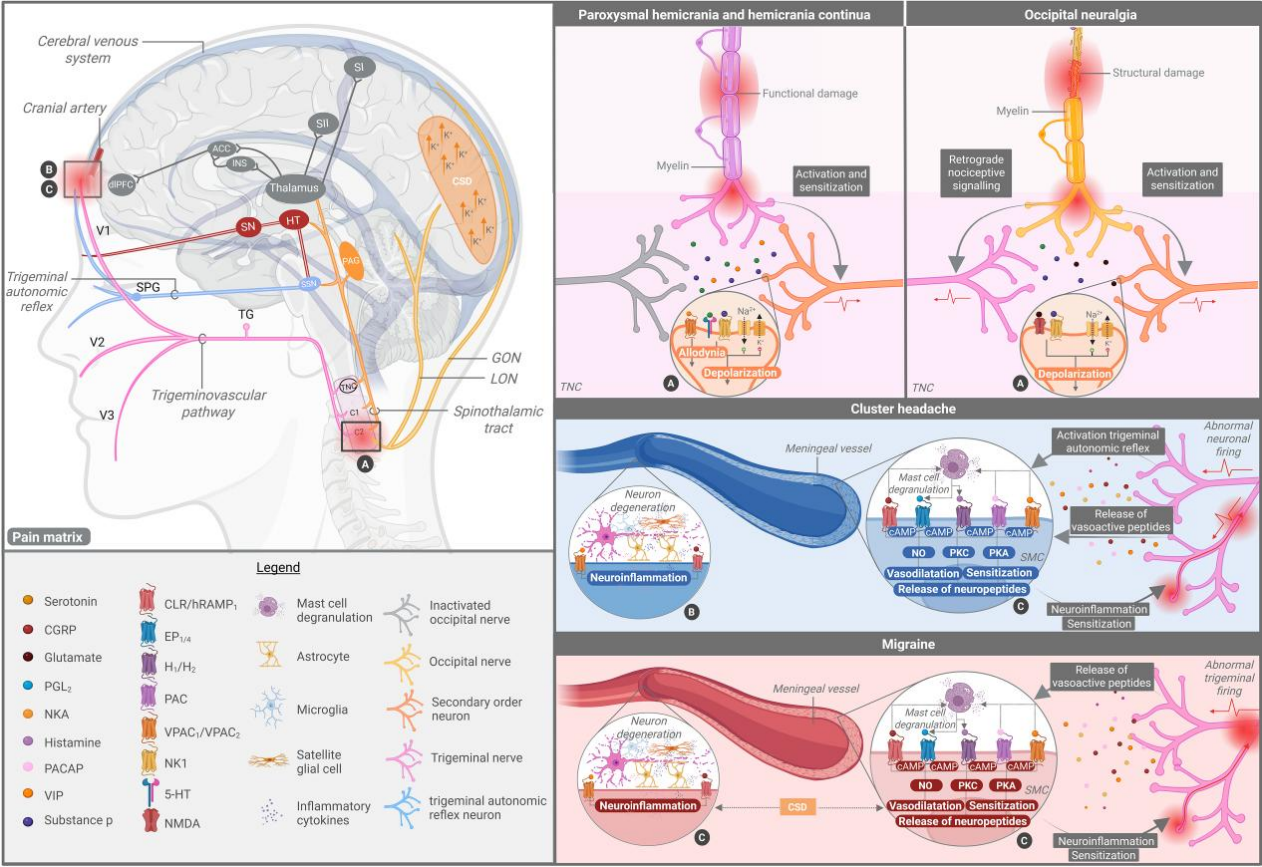
**Molecular mechanisms in headache pathology.** Overview of molecular pathways in headache disorders, including trigeminal autonomic reflex and spinothalamic tract with corresponding molecules and receptors. With regard to the trigeminal autonomic reflex, it is hypothesized that irritation of the trigeminal nerve endings leads to activation of secondary order neurons originating from the trigeminal nucleus caudalis (TNC). This contributes to activation of parasympathetic efferents of the superior salivatory nucleus (SSN), projecting to the sphenopalatine ganglion (SPG). Fibres from the SPG innervate lacrimal, nasal and pharyngeal glands, triggering autonomic symptoms. The suprachiasmatic nucleus (SCN) of the hypothalamus is activated upon relay of signals from the retino-hypothalamic tract. As a result, the superior salivatory nucleus and TNC are activated, consequently contributing to autonomic symptoms and headache pain, respectively. Please note that (i) overlapping pathways may be present between headache disorders, and solely the most important differences have been depicted to assure clarity of the figure; and (ii) paroxysmal hemicrania (PH) and hemicrania continua (HC) are distinct diagnoses, and data from both conditions have been grouped in this figure. Therefore, caution is warranted when interpreting the results. ACC = anterior cingulate cortex; cAMP = cyclic adenosine monophosphate; CGRP = calcitonin gene-related peptide; CLR = calcitonin receptor-like receptor; CSD = cortical spreading depression; dlPFC = dorsolateral prefrontal cortex; EP = prostaglandin E2 receptor; GON = greater occipital nerve; H = histamine receptor; hRAMP = human receptor activity modifying protein; HT = hypothalamus; 5-HT = 5-hydroxytryptamine receptor; INS = insula; K = potassium; LON = lesser occipital nerve; Na^2+^ = sodium; NK1 = neurokinin-1 receptor; NKA = neurokinin A; NMDA = *N*-methyl-D-aspartate receptor; NO = nitric oxide; PAC = pituitary adenylate cyclase-activating polypeptide receptor; PACAP = pituitary adenylate cyclase-activating polypeptide; PKA = protein kinase A; PKC = protein kinase C; S1 = primary somatosensory cortex; S2 = secondary somatosensory cortex; SMC = smooth muscle cell; SN = subthalamic nucleus; TG = trigeminal ganglion; V1 = opthalmic branch; V2 = maxillary branch; V3 = mandibular branch; VIP = vasoactive intestinal polypeptide; VPAC = vasoactive intestinal peptide receptor. Created in BioRender. Kollenburg, L. (2025) https://BioRender.com/nji2p32.

The trigeminovascular pathway is a pathophysiological substrate in primary headache disorders.^22^ This pathway consists of trigeminal neurons innervating cranial and meningeal blood vessels. It is believed that disturbances in the trigeminal nerve and/or trigeminal nucleus caudalis (TNC) cause the release of various neuropeptides and neurochemicals, including substance P, calcitonin gene-related peptide (CGRP), pituitary adenylate cyclase-activating peptide (PACAP), vasoactive intestinal polypeptide (VIP) and nitric oxide (NO), leading to vasodilatation of cranial blood vessels and activation of nociceptors.^23,24^ Vasodilatation may trigger trigeminal nerve afferents, leading to enhanced nociception and signalling into the TNC, and consequently upstream activation of structures involved in pain processing^23,24^ (Fig. 2).

#### Migraine

Migraine manifests as throbbing unilateral pain in the frontal and/or temporal area, with attacks lasting between 4–72 h, associated with additional features like nausea/vomiting, photophobia, phonophobia and aggravation by routine physical activity.^7,25,26^ Patients with migraine may present with bilateral cranial autonomic symptoms (e.g. conjunctival injection, lacrimation, nasal congestion, eyelid oedema, forehead and facial flushing, aural fullness, miosis and ptosis), typically with one side being more prominently affected than the other.^27^ Furthermore, a third of patients experience migraine with aura, manifesting as visual, sensory, speech and language disturbances.^22,25^ Migraine can be categorized as episodic and chronic. Chronic migraine (CM) persists for ≥15 days/month, with at least eight migraine days/month for more than 3 months, whereas episodic migraine (EM) is defined as having <15 headache days/month.^7,25,28^ Prior to migraine headaches, prodromal symptoms like mood changes, neck stiffness, food cravings and frequent yawning can be present.^29^ Following the headache phase of migraine, patients can experience a postdrome, characterized by fatigue, mood changes and inability to concentrate.^30^

##### Headache pain

Various studies assess the involvement of several peptides in the pathophysiology of migraine and found altered levels in the CSF and plasma,^31-45^ as well as responses to pharmacological targeting^46^ and exogenous administration of specific peptides^33,47,48^ (Table 1). Early models of migraine suggested the involvement of NO, a potent vasodilator, in the occurrence of nociceptive sensations.^49^ Other studies add the involvement of CGRP to the pathophysiology of migraine.^33^ Data shows that CGRP is expressed on the C-fibres with its receptors being located on the Aδ fibres of the trigeminal ganglion, cranial blood vessels and TNC^40,50^ (Table 1). These fibres arise from the sensory trigeminal ganglion and project towards the cerebral and dural vessels^50,51^ (Table 1). It is hypothesised that vasoactive substances, including histamine, CGRP, PACAP, VIP and prostaglandin (PG), bind to receptors of smooth muscle cells, located within the meningeal artery, leading to vasodilatation and neuroinflammation by activation of protein kinases (PKA, PKG)^7,23,52^ (Fig. 2C and Table 1). Some hypothesize that vasodilatation activates trigeminal neurons, enhancing signalling into the TNC and contributing to neuronal sensitization and amplification of pain signals^53^ (Fig. 2). Others, however, view vasodilation as an epiphenomenon of migraine^54^ (Fig. 2).

**Table 1:** Overview of involved (neuro)peptides and neurochemicals in the pathophysiology of migraine, cluster headache, hemicrania and occipital neuralgia

(Neuro)peptide	Involvement in headache disorder^a^	Origin	Location of receptors	Concentration^b^	Role in pathophysiology
CGRP	M, CH, hemicrania, ON	Trigeminal nerve, dorsal ganglia, trigeminovascular system	Trigeminal ganglia, cerebral vessels, brainstem, dura mater	↑	Cranial vasodilation; (peripheral/central) sensitization; mast cell degranulation; CSD; photophobia; allodynia; NO-glutamine-dopamine siganling; glial activation; enhancing substance P release, initiating cAMP
NO	M, CH, hemicrania	Endothelial cells, smooth muscle cells	Cerebral vessels, trigeminal ganglia, brainstem	↑	Cranial vasodilation; neuroinflammation; sensitization
PACAP	M, CH	Hypothalamus and trigeminovascular system	Trigeminal ganglia, cranial arteries, hypothalamus, brainstem	↑	Cranial vasodilation; neuroinflammation; sensitization of neurons; photophobia; mast cell degranulation; allodynia
NTG	M, CH, hemicrania	External source	N/A	N/A	Cranial vasodilation; neuroinflammation; sensitization; trigger headache attacks
VIP	M, CH, hemicrania	Parasympathetic nerves, SPG	Cerebral vessels, brainstem, SPG	↑	Cranial vasodilation; mast cell degranulation; allodynia; proinflammatory and anti-inflammatory effects; interaction sympathetic and parasympathetic nervous system
Substance P	M, CH, hemicrania, ON	Trigeminal nerve, dorsal ganglia	Trigeminal ganglia, brainstem, cerebral vessels, dura mater	↑	Cranial vasodilation; mast cell degranulation; platelet aggregation; meningeal nociceptive receptor activation; plasma extravasation; cytokine expression; autonomic symptoms
GABA	M, CH	Interneurons in brainstem, thalamus, glial cells	Brainstem	↓	Reducing excitability of nociceptive neurons; modulating nociceptive input
Neurokinin A	M, CH, hemicrania	Trigeminal nerve, dorsal ganglia	Trigeminal ganglia, cerebral vessels, dura mater	↑	Cranial vasodilation; mast cell degranulation; platelet aggregation; plasma protein extravasion; inflammatory response
Neuropeptide y	M	Trigeminovascular system, autonomic nerves	Hypothalamus, trigeminovascular system, cerebral vessels	N/A	Cranial vasodilation; regulation of sleep; stress response; promotion of feeding
Orexin	M	Hypothalamus	Hypothalamus, brainstem, thalamus	=/↓	Modulation structures involved in pain processing; enhance neuronal response to nociceptive stimuli; appetite
Serotonin	M, CH, hemicrania	Platelet, trigeminal nerve endings, Raphe nuclei in brainstem	Trigeminal ganglia, cerebral vessels, thalamus, cortex	↑	Vasoconstriction of cranial arteries and trigeminal nerve endings, affecting release of dopamine, acetylcholine and GABA
Glutamate	M, CH, ON	Trigeminovascular system, thalamus, cortical neurons	Trigeminal ganglia, brainstem, thalamus, cortical neurons	↑	Interaction with CSF; neuronal hyperexcitability
Dopamine	CH, M	Substantia nigra, hypothalamus	Hypothalamus, thalamus, brainstem	↑/↓	Preictal symptoms (migraine); rhythmicity of attacks (CH); autonomic symptoms; modulating pain
Interleukins	M, CH	Immune cells	Brainstem, cerebral vessels, trigeminovascular system	↑	Enhanced neuronal response via nodes of Ranvier
Histamine	M, CH	Mast cells, hypothalamus	Hypothalamus, cerebral vessels, cortical neurons, trigeminovascular system	↑	Vasodilatation of cranial arteries; sensitization of trigeminal nerve afferents; inducing prostaglandin release
Tryptophan	M	Peripheral and central neurons	N/A (converted into serotonin)	↓	Involved in migraine susceptibility
Melatonin	CH	Pineal gland	Hypothalamus, brainstem, retina, cerebral vessels	↓	Modulating nociceptive threshold; rhythmicity of attacks; regulating calcium influx of cerebral endothelial cells; enhancing effects of GABA
Somatostatin	CH	Hypothalamus, interneurons	Hypothalamus, cerebral vessels, trigeminovascular system	N/A	Inhibiting CGRP release

cAMP = cyclic adenosine monophosphate; CGRP = calcitonin gene related peptide; CH = cluster headache; CSD = cortical spreading depression; GABA = gamma aminobutyric acid; N/A = not applicable or not available; M = migraine; NO = nitric oxide; ON = occipital neuralgia; SPG = sphenopalatine ganglion; VIP = vasoactive intestinal peptide; ↑ = increased; ↓ = decreased; = = unchanged.

^a^Please note that the selected headache disorders in which certain peptides are mentioned to be involved were selected based on evidence from articles included within this review.

^b^Please note that ‘increased’ and ‘decreased’ refers to alterations in peptide levels measured during attacks of at least one of the headache disorders.

CGRP is also thought to have a direct effect on the glutamatergic system by enhancing synaptic transmission mediated by glutamate, leading to activation of the trigeminovascular system and thalamus.^24,55,56^ Furthermore, CGRP potentially activates astrocytes, microglia and satellite glial cells of the trigeminal ganglion^23^ (Fig. 2C). They release proinflammatory cytokines such as interleukins (IL-1 and IL-6) and tumour necrosis factor-alpha (TNF-α), leading to enhanced neural response by acting upon the nodes of Ranvier^41,53,57-59^ (Table 1). This theory is supported by authors reporting reduced trigeminal hyperalgesia with lower levels of CGRP.^60,61^ Literature suggests that CGRP also enhances the effects of NO, as it influences the release of NO and corresponding metabolites and acts upon similar mechanisms of vasodilatation^62-64^ (Table 1). Other reports mention that CGRP has a role in migraine by contributing to mast cell degranulation and initiation of cyclic adenosine monophosphate (cAMP)^56^ (Fig. 2C and Table 1). Indirect effects of CGRP in migraine, such as enhancing the release of substance P from small-diameter ganglion cells, resulting in activation of meningeal nociceptive receptors and plasma extravasation in the dura mater, are also reported.^65,66^ Enhanced release of substance P is thought to be involved in vasodilation of cranial arteries, mast cell degranulation, and platelet aggregation, consequently contributing to the pathogenesis of migraine^33^ (Table 1).

PACAP is thought to contribute to mast cell degranulation in the dura mater.^67^ Mast cell degranulation causes the release of prostaglandins and histamine, which have comparable effects on vascular endothelium as CGRP and PACAP^68^ (Fig. 2C). Histamine is also thought to interact with mast cells and CGRP, causing sensitization of trigeminal nerve afferents in migraine sufferers^69^ (Table 1). Similar to CGRP, exogenous nitroglycerin (NTG) is reported to play an important role in migraine, as it can be converted to NO in the endothelial wall, consequently contributing to triggers of migraine headaches.^70^ Aside from NTG, altered metabolism of tryptophan is linked to migraine susceptibility, as well as development and continuation of attacks^61^ (Table 1). Another neurotransmitter to be discussed is gamma-aminobutyric acid (GABA), which is released from interneurons in the dorsal horn. It is thought to reduce the excitability of nociceptive neurons via presynaptic inhibition.^71^ GABA is therefore suspected to be involved in terminating migraine attacks (Table 1). With regard to the triggering of headache attacks, VIP, a neuropeptide with vasodilatory properties, is thought to be involved.^48^

Various PET studies have also pointed towards the potential involvement of neuroinflammatory markers such as translocator protein (TSPO) in migraine pathology.^72-75^ More specifically, TSPO is a marker of glial activation, which is often upregulated in neuroinflammatory states. Studies found significant brain elevations in ^11^C-PBR28, a radioligand that binds to TSPO, suggesting that neuroinflammation plays a role in migraine pathology.^72-75^ Interestingly, while several studies suggest a potential association between headache frequency and neuroinflammation,^75^ the nature of this relationship remains inconsistent, as some report a positive correlation,^72^ while others describe a negative one.^73^ Other PET studies focused on opioid and dopamine receptor availability in patients with migraine.^76-78^ Authors report an overall decrease in mu-opioid receptor binding potential non-displaceable (μOR BP_ND_), a selective measure for availability of the μ-opioid receptor, in limbic structures of patients with migraine compared to healthy controls.^76,77^ It is hypothesized that altered opioid signalling contributes to headache pain in migraine through the induction of hyperalgesia/allodynia.^76^ This theory is supported by outcomes showing that the use of opioids can produce opioid-induced hyperalgesia when used to treat migraine.^79^ The promising results of opioid-targeted therapies also reinforce the involvement of the opioid system in migraine pathology.^80,81^ Several articles mention that increased endogenous opioid signalling may be modulated by the attack frequency, intensity and duration, thereby raising the question of whether alterations in opioid signalling are a cause or a consequence of migraine.^76,77^ Aside from opioid signalling, altered dopamine receptor availability has also been observed in migraine patients, with distinct D2/D3 receptor patterns that can differentiate them from healthy controls.^78^ Authors report that dopamine receptor hypersensitivity is a key hallmark of migraine,^82^ a notion supported by clinical findings demonstrating increased sensitivity to dopamine agonists and therapeutic improvement with dopamine antagonists in migraine patients.^83^ Altered dopaminergic signalling in migraine is thought to contribute to headache pain through modulation of the trigeminovascular system, a key component in migraine pathology.^83^

##### Associated and autonomic symptoms

Associated symptoms of migraine, like altered sleep and appetite, are linked to the release of neuropeptide Y (NPY), leading to vasoconstriction and activation of the orexigenic pathway in the hypothalamus^84-86^ (Table 1). Orexin is a neuropeptide synthesized in the lateral hypothalamus, with widespread projections across the CNS, including various areas of the brainstem that are involved in nociceptive processing.^85^ Although its exact role in migraine is yet to be elucidated, orexin may enhance neuronal responses upon nociceptive stimulation.^85^ It is also thought to interact with the affective component of pain, via the orexigenic-noradrenergic pathway, reaching the amygdala, consequently affecting mood, awareness and the way patients deal with their symptoms^85^ (Table 1). NPY and orexin are both associated with regulating appetite; hence, they may also play a role in food cravings, which are often experienced in the prodromal phase.^33,85^ Another key modulator of symptoms seen prior to attacks is dopamine, which is found to be elevated during symptoms of nausea, vomiting and dizziness in patients with migraine relative to controls.^83^

With regard to autonomic symptoms of migraine, the sphenopalatine ganglion (SPG) is thought to be an important site for the interaction between sensory and parasympathetic pathways through VIP signalling.^87^ VIP is released from parasympathetic fibres within the SPG. Upon binding to its receptors, it could lead to a cascade of events, causing vascular and autonomic symptoms associated with migraine^48^ (Table 1). VIP is also found in parasympathetic nerves innervating cranial blood vessels, thus connecting the sensory and parasympathetic system, leading to associated symptoms like nausea and vomiting.^33^ Another potential theory underlying autonomic symptoms in migraine includes sensitization of the sympathetic nervous system through upregulation of postsynaptic neurotransmitters (e.g. acetylcholine, CGRP and VIP) and repeated stimulation by the sensory system.^88^ Additionally, disrupted GABA inhibition in the locus coeruleus and periaqueductal grey (PAG) by extensions are thought to facilitate the transmission of sensory signals and initiate activation of the sympathetic autonomic systems.^89^

##### Aura

An important phenomenon that is believed to be associated with aura in migraine is cortical spreading depression (CSD), which refers to a slow wave of neural and glial depolarization followed by sustained underactivity^66^ (Fig. 2). CSD is initiated by a massive increase in extracellular potassium and glutamate and reduced levels of sodium and calcium, consequently leading to expression of the c-Fos protein, a marker of neuronal activity, into the TNC.^23,90-94^ It also enhances the release of substance P, neurokinin A (NKA) and CGRP into the TNC and trigeminal nerve terminals^23,90-94^ (Table 1). There appears to be a bidirectional correlation between glutamate and CSD, as both seem to enhance the effects of one another.^23^ Plasma glutamate levels are also found to be higher in patients with migraine, and are suspected markers of neuronal hyperexcitability, which is hypothesized to play a role in aura.^71^ Furthermore, PACAP and NO are defined as potential triggers of aura in migraine due to their effects on functional brain networks.^33,95^ In addition, reduced serotonin (5-HT) release is thought to influence aura by activation of the trigeminovascular nociceptive pathway.^96,97^ Lastly, 5-HT is thought to be involved in the release of dopamine, acetylcholine and GABA, thus interacting with other neuropeptides involved in migraine pathophysiology. High 5-HT levels are theorized to have a positive effect on preventing migraine attacks and aura, as it may cause desensitization of the 5-HT receptor^98^ (Table 1).

#### Cluster headache

CH is characterized by extremely severe unilateral pain in the temporal or periorbital region, often accompanied by restlessness and autonomic symptoms like lacrimation, eyelid oedema, conjunctival injection, miosis, ptosis and/or nasal congestion.^7,8,26,99,100^ CH attacks last approximately 15–180 min, with episodes occurring up to eight times/day for weeks to months, with a remission of months to years in most patients.^8,100,101^ Periods in which patients experience CH attacks are referred to as ‘bouts’. When bouts continue ≥1 year without remission periods of up to three consecutive months, it is referred to as chronic CH (CCH); otherwise, it is defined as episodic CH (ECH).^99^ Attacks often follow a circadian rhythm, with bouts frequently showing a higher incidence during the spring and autumn.^102-105^ In CH, three vital components are hypothesized to be involved in the clinical symptoms: the hypothalamus, trigeminovascular pathway, and trigeminal autonomic reflex^103,106,107^ (Fig. 2).

##### Headache pain

Various signalling molecules, including IL-2 and NO, as well as neuropeptides such as VIP, CGRP, dopamine, glutamate, histamine and PACAP, are reported to be elevated during CH attacks and reduced afterwards.^101,108-120^ In addition, these signalling molecules and neuropeptides are hypothesized to play a role in the trigeminovascular pathway and trigeminal autonomic reflex of CH.^108-119^ Upon activation of the trigeminal autonomic reflex, vasodilatory molecules like CGRP, VIP, NKA and PACAP are released from the trigeminal ganglion^106^ (Table 1 and Fig. 2C). Studies investigating CGRP and CH, found that this peptide may interact with platelets by impairing dopamine degranulation, leading to elevated dopamine levels and sensitization of cortical areas involved in pain perception^121,122^ (Table 1). Dopamine, on the other hand, is associated with pain modulation, autonomic functions and hypothalamic activity^111^ (Table 1). Similar to migraine, CGRP also activates mast cells in CH, causing neurogenic inflammation, and acts as a vasodilator, leading to activation of downstream kinases and release of NO^109,123,124^ (Table 1 and Fig. 2A). Notably, disease activity of CH and release of CGRP have been linked to each other, as CGRP release is higher in patients with remission compared to those having CCH. A plausible explanation for this could be depletion of CGRP in the trigeminal fibres of patients with CCH.^125^ Histamine also leads to activation of kinases in CH, via induction of prostaglandin release in mast cells, eventually contributing to sensitization of trigeminal nerve afferents^109,126^ (Table 1 and Fig. 2C). Data also show lower levels of somatostatin during and between bouts of CH, which may contribute to the pathophysiology by inhibiting CGRP release.^125^

Glutamate and PACAP are thought to enhance firing into the suprachiasmatic nucleus (SCN), leading to melatonin release from the pineal gland and initiation of CH attacks.^106,125,127^ Melatonin is thought to reduce the nociceptive threshold by regulating calcium influx of cerebral endothelial cells, enhancing the effects of GABA and modulating 5-HT receptor activity^103^ (Table 1). The involvement of 5-HT is bolstered by reports showing positive effects of sumatriptan, a 5-HT1b,d receptor agonist, and altered expression of this receptor in patients with CH.^46,128^ Release of GABA from neurons and glial cells may also contribute to CH attacks, as it is thought to modulate nociceptive transmission in the TNC through GABA_A_-receptors^129-131^ (Table 1). Important to note is that kynurenine metabolism, a biochemical pathway involving the breakdown of the amino acid tryptophan, is also found to be altered during attacks and bouts, and is thought to contribute to the sensitization of neurons and altered glutamate transmission in CH.^132-134^ Similar to migraine, it is described that NTG may trigger CH attacks.^135^

##### Autonomic symptoms

An important mechanism contributing to autonomic symptoms of CH are the autonomic efferents from the SPG in the trigeminal autonomic reflex,^136-139^ which release NO, VIP, CGRP and PACAP, leading to activation of certain glands (e.g. nasal, salivary and lacrimal gland) and vasodilation in cranial blood vessels located near the temporal and orbital region^106,140-142^ (Table 1 and Fig. 2). Release of CGRP and VIP during trigeminal-parasympathetic discharge is thought to induce the release of mast cells, likely contributing to inflammation near cranial blood vessel leading to autonomic symptoms in CH.^114^ With regard to miosis, sialorrhea and nasal congestion, release of substance P via the trigeminal nerve into the iris and salivary and nasal glands is thought to be involved.^143^ Postganglionic fibres from the cervical sympathetic pathway project to peripheral target sites (e.g. eyes and forehead) and form a plexus around the carotid artery. Owing to the anatomical arrangements of the cervical sympathetic system, it is suspected that suppression of the sympathetic plexus around the internal carotid artery contributes to oculo-sympathetic deficit, flushing and sweating on the symptomatic side of patients with CH.^114^

#### Paroxysmal hemicrania and hemicrania continua

PH and HC manifest as extremely severe unilateral pain in the temporal or supraorbital area and are often accompanied by restlessness and autonomic symptoms like nasal congestion or rhinorrhoea, miosis, eyelid oedema, ptosis and conjunctival injection.^7,26,144-147^ In PH, headache attacks last for 2–30 min, occurring with a frequency of >5 attacks/day.^147^ Episodic PH is characterized by ≥2 bouts lasting from 7 days to 1 year when untreated, separated by a remission period of ≥3 months. If there is no remission period or if the remission duration is <3 months for at least a year, patients are said to have chronic PH. In HC, pain is present for more than 3 months and is often accompanied by exacerbations of moderate or greater intensity.^144,145^ Both PH and HC are responsive to therapeutic doses of indomethacin.^144^

##### Headache pain and autonomic symptoms

Various theories have been proposed for the pathways involved in PH and HC, including vasodilatation of intracranial arteries and dysregulation of the trigeminal autonomic system.^148-152^ Neuropeptides including 5-HT, substance P, NKA, NO, CGRP and VIP are thought to play an important role in these processes^148,153,154^ (Table 1 and Fig. 2A). Similar to other primary headache disorders, disturbances in the trigeminal pathway of patients with PH and HC may lead to the release of these peptides.^154^ These peptides can bind to receptors on the secondary order neuron of the TNC (Fig. 2A), the trigeminal terminals and ganglion, and smooth muscle cells located on intracranial arteries, leading to release of NO, vasodilatation, platelet aggregation and mast cell degranulation^153,155^ (Table 1). This cascade of events triggers nociceptive fibres, causing headache pain.^155^ The involvement of these peptides is supported by studies reporting elevated levels of CGRP and VIP in the jugular bulb of patients with PH and HC, which decrease to normal levels after successful treatments.^156-158^ Another interesting finding is the reduction in pain threshold, although this is more likely a consequence rather than a cause of PH and HC.^155^ Although circadian or circannual rhythmicity is less prominent in PH and HC attacks, it is suggested that the hypothalamus plays an important role in regulating headache pain and autonomic symptoms by initiating the trigeminal autonomic reflex.^101,156,159-161^ Though not directly measured in patients with PH and HC, it is suspected that administration of NTG may also be involved in triggering headache attacks^162^ (Table 1). With regard to sympathetic symptoms like miosis and ptosis, it is suggested that the release of vasoactive neuropeptides causes swelling of the carotid wall, resulting in the suppression of sympathetic nerve fibres supplying the pterygopalatine fossa, consequently leading to a lack of sympathetic tone.^101,156,163^

#### Occipital neuralgia

Symptoms of ON include sharp or shooting pain in the nuchal-occipital distribution, which can last seconds to minutes, and be severe in intensity.^7,164,165^ Pain occurs repeatedly and starts unilaterally, but may be bilateral.^164,166^ ON is also characterized by tenderness, allodynia and/or dysaesthesia in the affected area.^166^ The main pathophysiological mechanism is thought to be compression or injury and/or trauma to the occipital nerve.^167^ The occipital nerve consists of various branches, including the greater occipital nerve (GON), lesser occipital nerve (LON) or third occipital nerve (TON), all of which may be involved in ON^8,168,169^ (Fig. 2).

##### Headache pain and sensitization

Throughout the years, studies have proposed detailed mechanisms explaining how compression and/or trauma of the occipital nerve leads to pain.^170-174^ Trauma and/or compression causes increased release of various substances like glutamate, and substance P into the TNC^171^ (Fig. 2A). As a result, depolarization is initiated across the membrane of second-order nociceptive neurons, inducing activation of the spinothalamic tract and higher cortical areas involved in pain processing, leading to the development of allodynia and headache pain in the occipital area^175^ (Table 1 and Fig. 2A). In some patients, referred pain towards frontal areas is present.^175^ Although referred pain in ON is a rare occurrence, with limited data explaining this phenomenon, it appears that the close connections between the occipital nerve and trigeminovascular system, emerging in the TNC, are involved.^176^ These connections may be responsible for conducting retrograde nociceptive signalling from the occipital nerve to the trigeminal nerve via the TNC^177^ (Fig. 2A). This possibly also forms an explanation for the co-existence of ON and migraine.^171,178^ Other theories for referred pain following GON entrapment include dysfunction of descending pain modulating pathways, and sensitization of secondary neurons in the TNC and third-order neurons in the thalamus.^175^ Hyung *et al*.^175^ report that the release of glutamate, CGRP, PACAP and cytokines from glial cells is involved in central sensitization (Table 1 and Fig. 2A). The occurrence of sensitization and hypersensitivity in ON is thought to be related to constant compression and/or vascular contact with the occipital nerve, leading to continuous nociceptive signalling into the TNC.^176,177^ Consequently, this enhances the release of glutamate and substance P from the trigeminal nerve, and/or reduces local segmental spinal inhibition.^171,179^ Over time, these processes are thought to cause long-term potentiation in neurons of the TNC, leading to hypersensitization and/or allodynia^169^ (Table 1). This hypothesis is bolstered by studies showing enhanced convergence of dural and cervical afferents in the TNC upon stimulation of the GON.^180^ Another theory explaining sensitization in ON is impairment in brainstem pain-modulatory structures.^171,175^

### Regional activity patterns and connectivity pathways

Advances in functional neuroimaging techniques, such as functional MRI (fMRI) and single-photon emission computed tomography (SPECT), have enabled a deeper understanding of the underlying pathophysiology of headache disorders.^28^ Studies have shown the presence of atypical functional activity (FA) and functional connectivity (FC) in various cortical and subcortical structures and their connections.^181^

#### Migraine

Studies investigating FA and FC in individuals with migraine perform neuroimaging in different phases. Migraine consists of four distinct phases, starting with the preictal phase (prodromal phase), followed by the aura phase, which is the time period prior to headache attacks, and subsequent to the ictal phase—the period during which patients experience the actual headache. The last phase is the postictal phase, also known as postdrome, referring to the time following the headache.^30^ The interictal phase refers to the period between migraine attacks.^30^

##### Interictal, preictal and ictal phase

**Figure 3:**
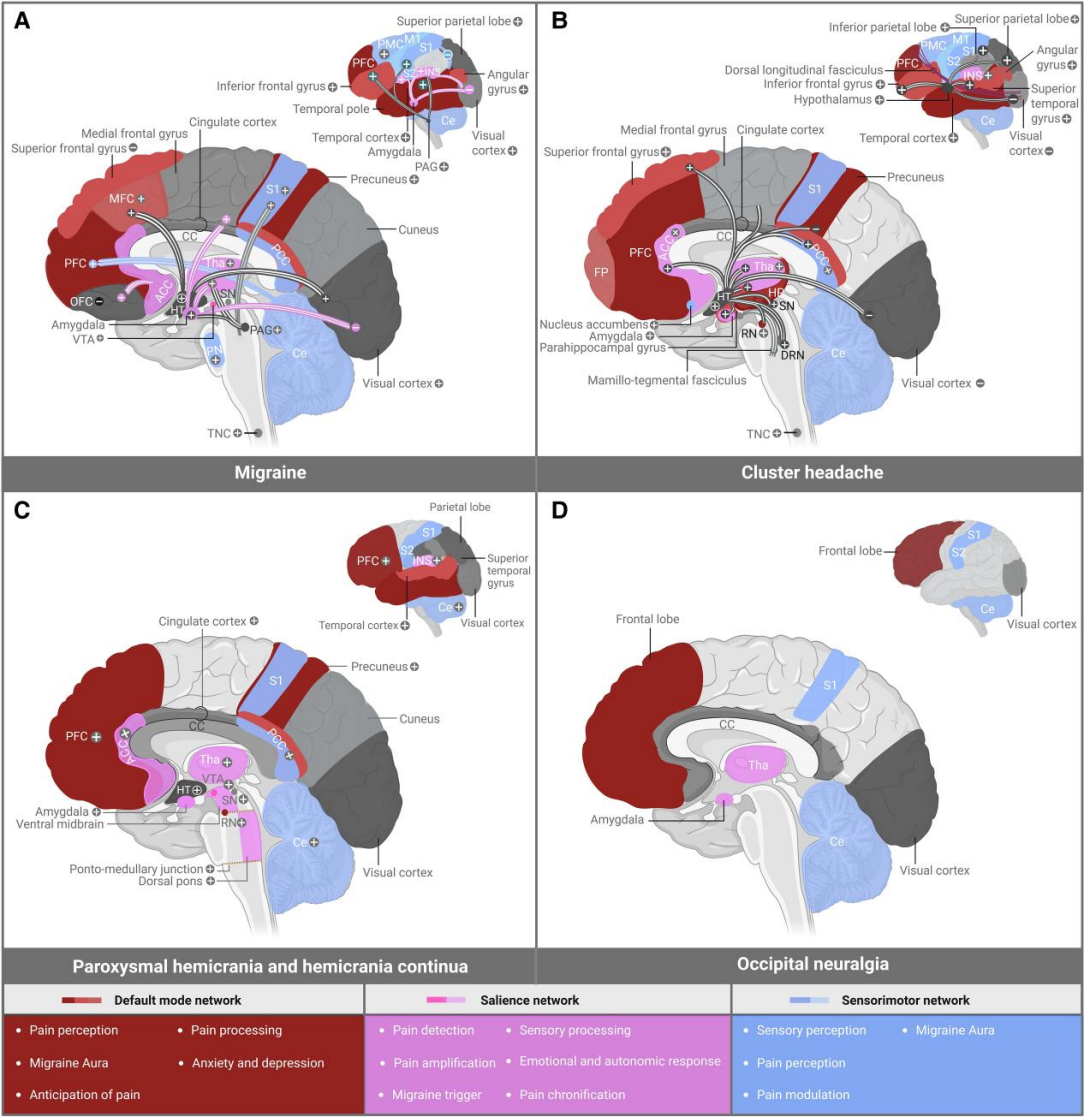
**Regional activity patterns and connectivity pathways in headache pathology.** Overview of connectivity changes in migraine (**A**), cluster headache (**B**), paroxysmal hemicrania and hemicrania continua (**C**) and occipital neuralgia (**D**). Structures and functions of the default mode network, salience network and sensorimotor network are depicted. + = enhanced activity; − = reduced activity; ACC = anterior cingulate cortex; CE = cerebellum; DRN = dorsal raphe nucleus; FP = frontal pole; HP = hippocampus; HT = hypothalamus; IFG = inferior frontal gyrus; INS = insula; M1 = primary motor area; MFC = medial frontal cortex; OFC = orbitofrontal cortex; PAG = periaqueductal grey; PCC = posterior cingulate cortex; PFC = prefrontal cortex; PMC = premotor cortex; RN = red nucleus; S1 = primary somatosensory cortex; SMA = supplementary motor area; SN = substantia nigra; Tha = thalamus; TNC = trigeminal nucleus caudalis; VTA = ventral tegmental area. Created in BioRender. Kollenburg, L. (2025) https://BioRender.com/ba7flvx. *Please note that PH and HC are distinct diagnoses, and that data from both conditions have been grouped in this figure. Therefore, caution is warranted when interpreting the results.

Imaging studies report differential FC characteristics in the executive and central and dorsal attention (DAN),^182,183^ default mode (DMN), central executive network (CEN)^184,185^ and sensorimotor network (SMN)^186,187^ during various phases of migraine.^188^ Starting with the interictal phase, increased FC of the PAG with right ventrolateral prefrontal cortex (PFC), insula, thalamus, parahippocampal gyrus, amygdala, secondary somatosensory cortex (S2), angular, occipital, temporal and parietal cortex, parietal operculum, and lingual and marginal gyrus is found alongside decreases in FC of the insula, and medial, lateral, dorsomedial and superior PFC^189-195^ (Fig. 3A, Table 2 and Supplementary Table 1). Notably, both decreased^190^ and increased^194,205^ FA of the anterior cingulate cortex (ACC) are reported, likely pointing towards heterogeneity in the functioning of the cingulum. Considering that the cingulum is a central hub connecting various areas involved in pain processing,^206^ it may be theorized that the ACC aids in regulating the interictal phase by altering FC to higher cortical areas involved in pain. Another interesting finding is the negative correlation between FC to the middle frontal gyrus and the intensity of migraine attacks.^190,207^ Regarding the lingual gyrus, it is thought to interfere with migraine phases as increased FC is found in various patients.^120^

**Table 2:** Overview of functional and structural changes in migraine

Author	Study design^a^	Number of patients	Diagnosis	Experimental paradigm	Phase of measurement	Imaging modality	Results
Karsan *et al*.^196^	I, LS	*n* = 21 (M)	MwA (*n* = 11) and MwoA (*n* = 10)	Triggered attacks with nitroglycerin (*n* = 21) and placebo administration (*n* = 21)	During headache and preictal phase	rs-fMRI	Baseline versus triggered attack in MwA/MwoA: increased FC of the pons with the cerebellar tonsils, medulla and limbic cortical areas. Decreased FC between pons and limbic cortical areas Baseline versus triggered attacks and placebo versus nitroglycerin in MwA/MwoA: decreased thalamic connectivity with cuneus and precuneus in nitroglyceirn group
Schulte *et al*.^197^	O, LS	*n* = 8 (M)	EMwA (*n* = 1) and EMwoA (*n* = 7)	N/A	Ictal, interictal and preictal phase	rs-fMRI	Baseline versus attacks in EMwA/EMwoA: increased FC between pons with hypothalamus and nucleus accumbens
Veréb *et al*.^198^	O, CC	*n* = 57 (M) *n* = 32 (HCo)	MwA (*n* = 20) and MwoA (*n* = 37)	N/A	Interictal period	rs-fMRI	MwA versus MwoA: increased FC between right insula and dorsal ACC MwA versus HCo: increased FC between dorsal ACC and left PFC MwoA versus MwA and HCo: decreased FC between right insula and PFC
Maleki *et al*.^199^	I, LS	*n* = 19 (M)	EMwA and EMwoA	Painful heat stimulation of the hand	Ictal and interictal phase	fMRI	Noxious stimulation in no allodynia versus generalized allodynia in EMwA/EMwoA: increased trigeminal nucleus and thalamus, but no differences in FA trigeminal ganglion Ictal versus interictal phase in EMwA/EMwoA: alterations in brainstem/pons, thalamus, insula, cerebellum and cingulate cortex
Martinelli *et al*.^200^	I, LS	*n* = 5 (M)	EMwoA	Triggered attacks with nitroglycerin	Pain-free condition, prodromal and full-blown phase of attack	rs-fMRI	Baseline versus attack in EMwoA: decreased FC thalamus and pons, orbital gyrus, cerebellum
Van Oosterhout *et al*.^201^	I, LS, CC	*n* = 12 (M), *n* = 10 (HCo)	EMwoA	Attacks triggered with nitroglycerin (*n* = 12)	Preictal and interictal phase	fMRI	Baseline versus triggered or spontaneous attacks in EMwoA: increase in hypothalamic activity in triggered and spontaneous attacks Baseline versus attacks in HCo: no functional changes
Cao *et al*.^202^	O, LS, CC	*n* = 44 (M), *n* = 44 (HCo)	MwoA	N/A	Interictal phase, during an attack	rs-fMRI	MwoA versus HCo: decreased grey matter in middle frontal gyrus MwoA with headache versus HCo: increased FC between middle frontal gyrus and cerebellum MwoA interictal phase versus HCo: decreased FC in cingulum, superior frontal gyrus and middle frontal gyrus and precuneus Interictal versus during attacks in MwoA: decreased FC between middle frontal gyrus and superior frontal gyrus
Gollion *et al*.^203^	O, CC	*n* = 21 (M), *n* = 18 (HCo)	MwA	N/A	Interictal	rs-fMRI	MwA versus HCo: enhanced FC of the right and left anterodorsal insula and clusters in the upper cerebellum
Porcaro *et al*.^204^	O, CC	*n* = 15 (M), *n* = 20 (HCo)	EMwoA	N/A	Ictal phase	DTI and rs-fMRI	EMwoA versus HCo: decreased fractional anisotropy of hypothalamus

ACC = anterior cingulate cortex; CC = case control study; DTI = diffusion tensor imaging; EM = episodic migraine; FA = functional activity; FC = functional connectivity; fMRI = functional MRI; HCo = healthy controls; I = interventional study; LS = longitudinal study; M = migraine; MRI = magnetic resonance imaging; N/A = not available or not applicable; O = observational study; PFC = prefrontal cortex; rs-fMRI = resting-state MRI; wA = with aura; woA = without aura.

^a^Please note that other review articles are not included within this table and that this table presents a shortened version of Supplementary Table 1, displaying only a subset of the most recent studies.

For the preictal phase, increased FA and FC in the hypothalamus, tegmental area, PAG, pons, occipital, temporal and PFC have been reported when compared to the ictal phase.^196,201,208,209^ Added to that, FC of the pons with limbic cortical areas appears to be decreased.^196^ fMRI outcomes are supported by PET studies showing altered cerebral blood flow (CBF) in a subset of these areas^37^ (Fig. 3A and Supplementary Table 1). It is suspected that photophobia and hypersensitivity, present during the preictal phase, are linked to abnormal processing within thalamic structures, as it forms a relay for ascending nociceptive information from the brainstem to various cortical regions.^188,210^ These outcomes may point towards a potential role of mesolimbic structures in symptoms associated with the preictal phase, especially when considering its involvement in mood, attention, reward and sensory processing.^211^

Regarding the ictal phase, increased FC with the frontal gyrus, PMC, medial frontal cortex (MFC), S2, hypothalamus and visual cortex, as well as decreased FC to the cerebellum, frontal lobe, and between DAN and CEN, is found^182,186,212-214^ (Fig. 3A and Supplementary Table 1). Studies comparing functionality in the ictal versus interictal phase note increases in FA of the pons, and FC between pons with hypothalamus, primary somatosensory cortex (S1) and nucleus accumbens, alongside decreased FC in the visual cortex and between middle frontal to superior frontal gyrus during the attack^197,199,200,202,215,216^ (Fig. 3A, Table 2 and Supplementary Table 1). Owing to the majority of studies finding brainstem abnormalities when comparing both phases, it may be theorized that this area plays a crucial role in switching from interictal to ictal phase.^217^ Nevertheless, findings by Meylakh and colleagues,^209^ suggesting that increased FA in the brainstem and hypothalamic regions during ictal and pre-ictal phases remains present between attacks, raise interest in other potential areas involved in switching between phases.

##### Chronic versus episodic migraine

Aside from different phases, imaging data also reveal a potential role of FC in the chronification of migraine. In patients with EM, decreased FC is present in the thalamus with S1 and PMC, occipital cortex and precuneus, insula, operculum and temporal lobe, when compared to healthy controls.^218,219^ The same group reports increased FC in the thalamus with primary motor and orbitofrontal cortex and parietal brain areas.^219^ For CM, decreased FC of the structures in the DMN, SN and CEN,^184,185^ including amygdala to occipital lobe,^220^ is mentioned relative to healthy controls (Supplementary Table 1). When comparing both forms, increased FC to the amygdala,^220^ insula, dorsolateral prefrontal cortex, thalamus and precuneus^221^ (Fig. 3A, Table 2 and Supplementary Table 1) is observed in CM relative to EM. Considering the importance of these areas in pain processing and the fact that most of these structures are part of the pain matrix, it may be theorized that this network is involved in the chronification of migraine. This hypothesis is strengthened by outcomes from Yu *et al*.,^222^ revealing a negative correlation of regional homogeneity in the pain matrix (rostral ACC) with duration of disease. Additionally, data showing an association between DMN abnormality and duration of migraine attacks may indicate a potential role of this network in the chronification of migraine.^183^ Due to the extensive involvement of the DMN in attention, dysfunction within this network may constrain switching away attention from painful stimuli, thus extending migraine episodes. Especially in the case of a hyperactive DMN, abnormalities may lead to prolonged changes in attack duration, and eventually chronification of migraine.^183^ Another theory suggests that increased and long-term peripheral and central sensitization of the trigeminal system is involved in the chronification of migraine.^223,224^

##### Aura

When comparing migraine sufferers without aura (MwoA) to healthy controls, increased FC of the ACC with orbitofrontal cortex, PFC,^207,225-227^ between the temporal pole and PFC,^228^ and the hippocampal gyrus and occipital cortex,^229^ as well as increased FC of the hypothalamus with precentral, parietal, temporal and frontal gyrus, brainstem areas and cerebellum^230^ are reported. Furthermore, diffusion tensor imaging (DTI) imaging reveals decreased fractional anisotropy in the hypothalamus of these patients.^204^ For migraine sufferers with aura (MwA), alterations include increased FC with the orbital, occipital, parahippocampal and temporal, frontal and lingual gyrus, cerebellum, as well as decreased FC with the insula, cingulate and frontal gyrus relative to healthy controls^203,229,231^ (Fig. 3A, Table 2 and Supplementary Table 1). Studies comparing MwA with MwoA found differences in FC with the right lingual and middle frontal, cingulate gyrus, occipital pole, precuneus,^195,229^ insula, temporal and occipital cortex.^198,229,232^ As most of these structures are part of the ‘pain matrix’, it is likely that abnormal processing of pain plays a major role in symptoms associated with aura. Owing to the fact that abnormalities in perfusion and thickness of the occipital cortex are seen in MwA,^233-235^ with visual symptoms being the most common clinical manifestation of aura,^210^ it seems likely that the occipital cortex plays a major role in regulating visual symptoms during aura. It has been argued that events like CSD may contribute to neuronal dysfunction, leading to the generation of aura in the human visual cortex. Furthermore, studies showing hyperresponsivity of the visual processing network following trigeminal stimulation suggest that altered functioning of the occipital cortex may also be caused by disturbances in the trigeminovascular system.^231^ This may imply that abnormalities in the occipital cortex are a consequence rather than a trigger of aura. The involvement of the visual cortex is further supported by evidence linking connectivity of the visual network to the severity of aura.^236^ However, reports revealing an absence of FA changes of the occipital cortex^233,237^ in MwA, alongside outcomes of increased FC in the visuospatial and medial visual cortex of MwoA,^238^ raise questions as to how the visual system contributes to the pathophysiology of aura in migraine.

#### Cluster headache

Neuroimaging studies in CH have measured FC in-bout and out-of-bout, and compared outcomes to healthy controls and between different subtypes of CH. In-bout refers to periods in which CH attacks occur frequently and out-of-bout is defined as the remission period in which the patient does not experience any attacks. The in-bout phase lasts between 6–12 weeks, and the out-of-bout phase, months to years.^239^

##### In-bout and out-of-bout

In-bout, increased FC of the hypothalamus with ventral tegmental area (VTA), dorsal raphe nuclei, substantia nigra (SN), red nucleus (RN) and subthalamic nucleus (STN) is found relative to healthy controls.^240^ Notably, a 2015 fMRI study by Qiu and colleagues^241^ reported reduced hypothalamic salience network coactivation in-bout compared to healthy controls, suggesting a potential role of this network in the pathophysiology. In addition, a review by Chong *et al*.^181^ analysed FC between attacks in-bout and reported increased FC to the ACC, posterior cingulate cortex (PCC), inferior parietal lobule, amygdala, parahippocampal gyrus, insula, PFC, thalamus, temporal cortex, occipital cortex, and angular gyrus.^120^ Considering the role of the PFC in higher-order cognitive functioning, enhanced FC in this area may amplify the body's response to nociceptive stimuli, thus contributing to the development of headaches.^242^ Others investigated CH out-of-bout and report decreased FC of the hypothalamus with the frontal and temporal-parietal pain control system,^243^ as well as altered FA in DAN,^244,245^ SMN, visual networks and pain matrix,^246,247^ suggesting distribution of abnormal informative processing of nociceptive signals^244,245^ (Fig. 3B). Interestingly, a correlation between these network abnormalities and duration of disease is reported, potentially directing towards these changes being a consequence rather than a cause of CH.^247^ Studies comparing both phases found decreased FC of the hypothalamus with medial frontal gyrus, precuneus and cerebellar areas,^248^ as well as increased FA in the ACC, basal ganglia, ipsilateral hypothalamus, frontal lobe and contralateral frontal cortex^249^ in-bout relative to out-of-bout (Fig. 3B, Table 3 and Supplementary Table 2). Notably, the strength of hypothalamic FC to the cerebellum is found to be correlated with the frequency of CH bouts,^248^ consequently contributing to growing interest in its potential involvement in initiating or terminating bouts.^181,255,256^ SPECT and PET studies also compare CBF out-of-bout CH with healthy controls and found either a presence^257-259^ or absence^260,261^ of differences (Supplementary Table 2).

**Table 3:** Overview of functional and structural changes in cluster headache

Author	Study design^a^	Number of patients	Diagnosis	Experimental paradigm	Phase of measurement	Imaging modality	Results
Qiu *et al*.^241^	O, CC	*n* = 21 (CH), *n* = 21 (HCo)	ECH	N/A	In-bout	rs-fMRI	ECH versus HCo: decreased hypothalamic SN coactivation.
Yang *et al*.^248^	O, LS, CC	*n* = 18 (CH), *n* = 19 (HCo)	ECH	N/A	In-bout and out-of-bout	rs-fMRI	ECH versus HCo: altered FC of hypothalamus with medial frontal gyrus and occipital cuneus In-bout versus out-of bout: decreased FC of hypothalamus with medial frontal gyrus, precuneus and cerebellar areas (tonsil, declive and culmen)
Arkink *et al*.^250^	O, CC	*n* = 103 (CH, M, CPH), *n* = 48 (HCo)	ECH (*n* = 24), CCH (*n* = 23), probable CH (*n* = 14), MwA (*n* = 14), MwoA (*n* = 19), CPH (*n* = 9)	N/A	N/A	VBM	ECH/CCH versus HCo: bilateral enlargement of the suprachiasmatic and paraventricular nuclei in ECH and CCH
Chou *et al*.^245^	O, LS, CC	*n* = 17 (CH), *n* = 18 (HCo)	ECH	N/A	In-bout and out-of bout	rs-fMRI	ECH versus HCo: increased FC in temporal, frontal, SN, DMN, somatosensory, DAS and visual network independently from bout period. in-bout versus out-of-bout: altered FC in the frontal and DAS
Faragó *et al*.^244^	O, CC	*n* = 17 (CH), *n* = 17 (HCo)	ECH	N/A	Out-of-bout	rs-fMRI	ECH versus HCo: increased FA in ipsilateral attention network and contralateral cerebellar network.
Ferraro *et al*.^240^	O, CC	*n* = 17 (CH), *n* = 16 (HCo)	CCH	N/A	In-bout	rs-fMRI	CH versus HCo: increased FC of ipsilateral posterior hypothalamus with VTA, dorsal raphe nuclei and bilateral substantia nigra, red nucleus and subthalamic nucleus. No differences in FC pf contralateral hypothalamus
Ha *et al*.^251^	O, CC	*n* = 10 (CH), *n* = 20 (HCo)	ECH	N/A	N/A	MRI	ECH versus HCo: increased strength and closeness of cingulate gyrus. Decreased volume of caudal ACC and postcentral gyrus
Chong *et al*.^243^	O, CC	*n* = 37 (M, CH), *n* = 22 (HCo)	ECH (*n* = 18), M (*n* = 19)	N/A	Out-of-bout	VBM	CH versus HCo and migraine: decreased FC of hypothalamus with frontal and temporal-parietal pain control system. No significant differences in hypothalamic region volume or total brain volume
Giorgio *et al*.^252^	O	*n* = 25 (CH, M)	ECH (*n* = 12), MwoA (*n* = 13)	N/A	Out of attack	rs-fMRI	CH versus migraine: increased FC within working memory and executive control networks
Möller *et al*.^253^	I, LS	*n* = 26 (HCo)	N/A	Administration of kinetic oscillation stimulation in the nostril	N/A	fMRI	Baseline versus during triggered autonomic symptoms: induced activation of brainstem and cerebellar regions and bilateral insular regions after nonpainful stimuli and enhanced FA of locus coeruleus, ventral posteriomedial nucleus of thalamus, anterior hypothalamus and ipsilateral insula following painful stimuli
Ferraro *et al*.^254^	I, CC	*n* = 28 (CH), *n* = 28 (HCo)	CCH	N/A	After CH attack	sMRI and rs-MRI	sMRI CCH versus HCo: Increased volume of bilateral nucleus accumbens, ventral diencephalon, hippocampus, frontal pole and right amygdala. Altered volume is present ipsilaterally to the pain ventral diencephalic regions and contralateral to the pain nucleus accumbens fMRI CCH versus HCo: reduced FC in right frontal pole-right amygdala pathway

ACC = anterior cingulate cortex; CC = case control study; CCH = chronic cluster headache; CH = cluster headache; CPH = chronic paroxysmal hemicrania; CR = case report; DAS = dorsal attention system; DMN = default mode network; DTI = diffusion tensor imaging; ECH = episodic cluster headache; FA = functional activity; FC = functional connectivity; fMRI = functional MRI; HCo = healthy controls; I = interventional study; LS = longitudinal study; M = migraine; M1 = primary motor cortex; MRA = magnetic resonance angiography; N/A = not available or not applicable; O = observational study; rs-MRI = resting-state MRI; sMRI = structural MRI; SN = salience network; VBM = voxel-based morphometry.

^a^Please note that other review articles are not included within this table and that this table presents a shortened version of Supplementary Table 2, displaying only a subset of the most recent studies.

Fluordeoxyglucose-PET imaging has revealed increased metabolism in the ACC, PCC, insula, thalamus and temporal cortex, alongside decreased metabolism in the cerebellopontine area and hypothalamus^262,263^ (Supplementary Table 2). In contrast, others report no significant differences when comparing metabolism,^264^ white blood cell uptake^265^ and density^266^ between these phases. Despite variability in outcomes, it seems likely that moving from out-of-bout to in-bout is characterized by an increase in abnormalities of these structural networks. Data showing correlations between strength of connectivity and frequency, as well as duration of CH attacks bolster this hypothesis.^181^

MRI studies comparing FC during versus out of a CH bout found increased FA of the ipsilateral hypothalamus, ACC, PCC, frontal temporal and parietal gyrus, amygdala, hippocampus and various brain stem areas, including the ipsilateral trigeminal root entry zone, bilateral RN and ventral pons.^267-269^ Interestingly, it is theorized that brain stem nuclei may contribute to CH attacks, despite activity in the RN usually being associated with pain avoidance.^246^ In addition, Qiu and colleagues^270^ report increased FC of the hypothalamus with various limbic structures (including ACC, PCC and amygdala) during versus out of CH attacks (Supplementary Table 2).

##### Chronic versus episodic cluster headache

Those investigating episodic CH (ECH)^250,252^ found increased FC of the hypothalamus with medial frontal gyrus and occipital cuneus,^248,271^ thalamus,^247^ temporal and frontal cortex, DAN, salience, DMN, attention and visual network^244,245^ compared to healthy controls. In addition, DTI and MRI studies also found higher mean diffusivity in frontal regions and reduced diffusivity in the limbic system,^272^ as well as decreased volume of the cingulate gyrus.^251^ For chronic CH (CCH), reduced FC in the right frontal pole-right amygdala pathway,^254^ increased FA of perigenual ACC, hypothalamus, thalamus,^268^ ACC and insula,^273^ hypothalamus with VTA, SN, RN and STN^254,274^ is mentioned (Fig. 3B, Table 3 and Supplementary Table 2). Most of these structures are part of the pain matrix and/or mesolimbic system, which leads to the theory that functional alterations in these networks are involved in the chronification of pain disorders like CH.^254^ The trigeminal and parasympathetic nuclei, on the other hand, may also contribute to the chronification as these are associated with sustaining CH.^103,275^ Important to note is that the theories regarding the chronification of CH are yet to be confirmed as only very few studies have been comparing FA and FC in ECH relative to CCH.

##### Rhythmicity and autonomic symptoms

The hypothalamus is assumed to play a major role in regulating rhythmicity of CH attacks and autonomic symptoms via its connections to the trigeminovascular and autonomic system.^269,270^ It is suspected that the hypothalamus is involved in igniting the trigeminovascular system during CH attacks and that afferents from the hypothalamus initiate the trigeminal autonomic reflex via the dorsal longitudinal fasciculus and mamillotegmental fasciculus^120^ (Fig. 3B). In favour of this theory are studies showing abnormalities in density,^122,266^ connectivity,^247,272^ CBF,^273^ metabolism^264^ and activity^269,273^ in the hypothalamus of patients with CH out-of-bout or out of attacks. Given its central location and connectivity to various cortical and subcortical areas, it seems likely that the hypothalamus plays a major role in rhythmicity of CH attacks^120,240,243,247,248,270,276^ (Fig. 3B). Leone *et al*.^101^ suggest that hypothalamic activity in CH could also give rise to a ‘central permissive state’, which allows for attacks to take place. Important to note is that, despite a potential central role of the hypothalamus in regulating CH, it is likely that a complex interplay between structures is responsible for initiating or terminating attacks, and that there is not a single ‘generator’.^120^ The brain stem, on the other hand, is suspected to be involved in pain avoidance and may, therefore, contribute to the fight-or-flight type behavioural pattern during attacks.^267^ It is suspected that interactions between the trigeminal autonomic reflex and various pain matrix areas contribute to autonomic symptoms in CH.^120^ This theory is supported by outcomes of Möller and colleagues,^253^ showing that provoked autonomic symptoms by administration of kinetic oscillatory stimulation in the nostril of healthy individuals induces activation of the brainstem, bilateral insula and cerebellum after nonpainful stimuli. Following a painful stimuli, enhanced FA of the thalamus, anterior hypothalamus and ipsilateral insula and locus coeruleus is observed (Table 2 and Supplementary Table 1). It however remains uncertain if these changes are a cause or consequence of nociceptive stimuli.

#### Paroxysmal hemicrania and hemicrania continua

##### Pain versus pain-free state

Imaging studies in PH and HC have been comparing FA and FC in pain to pain-free states.^277,278^ Matharu and colleagues report increased FA in the left and right PFC, thalamus, amygdala, insular cortex and anterior olfactory cortex^279^ in headache versus headache-free state. Further, PET and MRI studies show increased activation of the contralateral hypothalamus,^101^ ipsilateral dorsal rostral pons,^280^ ventrolateral midbrain, extending over the RN and SN,^278,281^ ACC, PCC, precuneus, cerebellum, postcentral gyrus and frontal and temporal cortex,^278,282^ as well as abnormalities in the cerebellum, superior temporal gyrus, corpus callosum, pituitary gland and parietal lobe, including S1 and S2,^283^ and fronto-temporal area^284^ (Table 4 and Fig. 3C). Altered brain stem activity, it is hypothesized to trigger PH and HC attacks.^278^ The brainstem receives input from the frontal cortex and hypothalamus, projecting towards the rostral ventromedial medulla, and may therefore be involved in initiating abnormalities in other brain areas of patients with PH and HC.^278^ The debate on brainstem activation being either pivotal or an epiphenomenon in PH and HC, however remains ungoing.^278^ Interestingly, other studies found no significant activation in the hypothalamus^281^ and other brain regions^282^ in painful versus pain-free state of patients with PH and HC. These outcomes may point towards the involvement of complex neuronal network deficit rather than a single structure in the pathophysiology of PH and HC.^279^ Notably, the use of indomethacin, a nonsteroidal anti-inflammatory drug, in various studies may also have contributed to differential outcomes in brain activity as solely in attack-free state (off indomethacin), no significant activations are found.^277,282^ It may be theorized that indomethacin improves headache symptoms by interfering with brain activity. Another explanation could be that in pain-free state (with indomethacin), similar structures are active, however below a threshold for pain perception, whereas in spontaneous termination of attacks, activity reaches further below that threshold.^282^

**Table 4:** Overview of functional and structural changes in hemicrania

Author	Study design^a^	Number of patients	Diagnosis	Experimental paradigm	Phase of measurement	Imaging modality	Results
Matharu *et al*.^278^	I, LS, CC	*n* = 7 (H), *n* = 7 (HCo)	HCwAS	Attacks treated with indomethacin or placebo/saline	Pain-free, painful period	PET	Painful period versus pain-free period in HC: significant activation of contralateral posterior hypothalamus and ipsilateral dorsal rostral pons. Activation of the ipsilateral ventrolateral midbrain, extending over the red nucleus and substantia nigra, S1, S2, and the bilateral pontomedullary junction. No intracranial vasodilatation Indomethacin versus placebo in HC: reduced activity in the dorsal rostral pons, posterior hypothalamus and ventrolateral midbrain following administration of indomethacin. These remained unchanged in placebo group. Indomethacin versus placebo in HCo: no significant alterations in activity following administration of placebo or indomethacin Indomethacin versus placebo in HC versus HCo: significant changes in contralateral posterior hypothalamus, ipsilateral dorsal rostral pons, ipsilateral ventrolateral midbrain region, ponto-medullary junction, ipsilateral thalamus, bilateral insulae, ipsilateral cingulate gyrus, PCC, bilateral frontal cortices including the contralateral precentral gyrus, contralateral postcentral gyrus, ipsilateral parietal cortex, bilateral temporal cortices, contralateral occipital cortex, ipsilateral cuneus, precuneus, and the cerebellum. No intracranial vessel dilatation was apparent.
Matharu *et al*.^284^	I, CS	*n* = 2 (H)	HCwAS	Attacks were treated with topiramate	N/A	MRI	In HC: encephalomalacia in the right fronto-temporal area in one patient, whereas no alterations were found in the other patient
Matharu *et al*.^282^	I, LS	*n* = 7 (H)	PHwAS	Pain-free periods were either spontaneous or induced with indomethacin	Pain-free, painful period	PET	Painful versus pain-free (off indomethacin): no regions of significant activations Painful versus pain-free (with indomethacin): significant activation in the posterior hypothalamus, contralateral ventral midbrain, ipsilateral lentiform nucleus, PCC, ACC, bilateral insula, bilateral frontal cortex, contralateral temporal cortex, contralateral postcentral gyrus, precuneus and contralateral cerebellum Pain-free (off indomethacin) versus pain-free (with endomethacin): increased activation in contralateral ventral midbrain, ipsilateral lentiform nucleus, anterior and posterior cingulate cortices, ipsilateral insulae, bilateral frontal cortices, contralateral temporal cortex, contralateral postcentral gyrus, precuneus and contralateral cerebellum
Valença *et al*.^280^	O, CR	*n* = 1 (H)	HC	N/A	N/A	MRI	In HC: no abnormalities in the hypothalamus
Irimia *et al*.^281^	I, CR	*n* = 1 (H)	HCwoAS	Attacks were treated with indomethacin	Pain-free, painful period	PET	Painful versus pain-free period: significant activation of dorsal pons. No activation in hypothalamus
Cittadini *et al*.^283^	I, CS	*n* = 39 (H)	HCwAS	Attacks were treated with indometacin	N/A	MRI or CT	In HC: abnormalities in frontal lobes, subcortical and paraventricular, operculum, cerebellum, superior temporal gyrus, corpus callosum, pituitary gland and parietal lobe^2^
Domitrz *et al*.^279^	I, CR	*n* = 1 (H, SUNCT, TN)	PHwAS, SUNCT, TN	Attacks were terminated with indomethacin	Pain-free period, painful period	fMRI	Painful versus pain-free period in PH: increased FA in left and right PFC, thalamus, amygdala, insular cortex, anterior olfactory cortex

ACC = anterior cingulate cortex; CC = case control study; CR = case report; CS = case series; CT = computed tomography; FA = functional activity; FC = functional connectivity; fMRI = functional MRI; H = hemicrania; HC = hemicrania continua; HCo = healthy controls; I = interventional study; LS = longitudinal study; N/A = not available or not applicable; O = observational study; PCC = posterior cingulate cortex; PFC = prefrontal cortex; PH = paroxysmal hemicrania; rCBF = regional cerebral blood flow; S1 = primary somatosensory cortex; S2 = secondary somatosensory cortex; SUNCT = short-acting unilateral neuralgiform headache; TN = trigeminal neuralgia; wAS = with autonomic symptoms; woAS = without autonomic symptoms.

^a^Please note that other review articles are not included within this table. The authors report that the majority of these findings are considered incidental.

##### Paroxysmal hemicrania versus hemicrania continua

Contralateral posterior hypothalamic activation is reported for HC and PH,^278,282^ whereas altered activation of the dorsal rostral pons is solely reported for HC^278,282^ (Table 4 and Fig. 3C). Based on these outcomes, it could be theorized that certain brainstem nuclei have an important role in the chronification of attacks as headache attacks persist longer in HC. Further, Matharu and colleagues describe patients with HC developing into chronic PH following treatment with cyclooxygenase-2 (COX-2) inhibitors.^150^ Considering the effects of COX-2 inhibitors on the hypothalamus,^150^ and neuroimaging studies showing altered hypothalamic activity^278,282^ in PH and HC, this structure could also potentially be involved in chronification of headaches.

##### Autonomic symptoms

There is a growing belief that the brainstem and hypothalamus play a crucial role in the development of autonomic symptoms.^278^ This idea arises from studies showing altered activity in these areas in patients with PH and HC and autonomic symptoms (HwAS) but not in those without autonomic symptoms (HwoAS) (Table 4). The brainstem contains antinociceptive and trigeminal nociceptive systems as well as autonomic regulatory centres, hence it is thought to contribute to autonomic symptoms in PH and HC.^280^ It is suggested that changes in the activity of brainstem centers^277,278,280,281^ are responsible for the trigeminovascular reflex as its connections could activate the parasympathetic outflow via the cranial nerve (VII) which regulates CBF.^280^ Moreover, it is theorized that cranio-autonomic symptoms are a result of central disinhibition of the trigeminal autonomic reflex by the hypothalamus via direct hypothalamic-trigeminal connections.^285^ Maestrini *et al*.^286^ report that hypothalamic activation in PH and HC either causes cranio-autonomic symptoms via hypothalamic modulation of the trigemino-vascular system or via direct output from the hypothalamus itself, pointing towards a more complex network leading to corresponding symptoms. Due to close proximity and widespread connections between the brainstem and hypothalamus,^156^ abnormalities in one of these structures may lead to altered activity in the other.

#### Occipital neuralgia

Neuroimaging of brain regions reveals that compression and/or trauma of the occipital nerve is likely a major cause of ON, which is supported by Yi and colleagues,^287^ who analysed the C2 roots in patients with ON using MRI. Functional imaging data of ON is very limited, however, a report focusing on stimulation of the occipital nerve, report decreased activity in the amygdala, bilateral primary visual, auditory and somatosensory cortex.^288^ Added to that, increased activity is observed in the bilateral thalamus, cerebellum, frontal and parietal areas^288^ (Fig. 3D). Though trauma and/or compression in ON is different from stimulation, both interfere with the functionality of the occipital nerve. For this matter, it may be theorized that similar alterations in brain functionality are present in patients with ON.

## Discussion

### Similarities and differences

#### Molecular mechanisms

Important mechanisms in the investigated headache disorders are central and peripheral sensitization, inflammation within the trigeminal system, and vasodilatation of cranial vessels^28^ (Table 1 and Fig. 2). Despite overlapping mechanisms, clinical manifestations (*e.g.* distribution of pain) vary among headache disorders. Pain can typically be present unilaterally or bilaterally in different regions including frontal/temporal for migraine, temporal or orbital for CH, PH and HC, and nuchal-occipital distribution for ON.^7^ These differences may be explained by variability in affected nerve branches, each innervating distinct anatomical areas (Fig. 3). The anatomical innervation of each of the affected nerve branches could also potentially explain autonomic symptoms like lacrimation, eyelid oedema, nasal congestion, miosis and/or ptosis in CH, PH and HC.^7^ The V1 and V2 branches of the trigeminal nerve innervate nasal glands leading to nasal congestion, as well as the ciliary body, eyelid, cornea and iris of the eye, contributing to symptoms like eyelid deem, ptosis and miosis.^289^

Interestingly, other disorder-specific traits like aura in migraine, rhythmicity of attacks in CH, and allodynia in ON may also be explained by the involvement of differential molecular pathways. With regard to rhythmicity and CH, melatonin and the hypothalamus may play a role in timing the attack, especially when considering that this phenomenon and altered melatonin levels^290^ are solely described for these patients (Table 1). This may suggest that the hypothalamus plays a more central role in CH compared to other pathologies, however this theory is yet to be proven. For migraine, CSD seems to be a well known phenomenon. CSD emerges within the occipital cortex and is thought to cause aura-like symptoms and activation of the trigeminal nociceptive system centrally and peripherally.^291^ Despite CSD being unique to migraine, it likely does not play a key role in initiating headache attacks as aura-like symptoms remain absent in two third of migraine patients.^181^ For allodynia, it becomes apparent that it involves multiple peptides, like CGRP, PACAP and VIP,^292,293^ which are altered in ON, but also in other headache disorders like migraine, PH, HC and CH (Table 1), hence pointing towards multifunctional peptides and a more complex mechanism reaching beyond these molecular pathways. Owing to the fact that autonomic symptoms are less prominent with no differences in vasoactive compounds like NO, NTG, VIP and NKA in patients with ON, it seems reasonable to assume that the trigeminal autonomic reflex and vascular system play an important role in migraine, CH, PH and HC, but less so in ON. Additionally, in migraine, CH, PH and HC, there is a primary dysfunction of the trigeminovascular system, whereas in ON, there appears to be dysfunction of this system only secondary due to potential retrograde nociceptive signalling from the occipital nerve to the trigeminal nerve^177^ (Fig. 3). This is likely another reason that may serve to explain differences in peptide levels and autonomic symptoms between these headache disorders. However, it should be noted that studies measuring levels of vasoactive compounds are very limited, especially for ON; hence caution should be taken in the interpretation. For this matter, there is an ongoing debate as to how the trigeminal system is involved in initiating headache attacks. Whereas some argue that trigeminal nerve disturbances are the main cause initiating attacks in these headache disorders, others suspect initiation by a more central component generating pain.^101,148^

Another notable difference between headache disorders are characteristics of pain as terms like ‘throbbing’ are used for migraine, whereas ‘intermittent piercing’ and ‘shooting’ forms of pain are mentioned for ON. This may be caused by the fact that in ON, there is an actual physical damage to the nerve^168^ whereas migraine involves functional nerve disturbances without structural damage (Fig. 3). Further, there is also variability in the duration and frequency of attacks among the investigated headache disorders. It is suspected that differences in frequency and speed of release and clearance of substances like CGRP and VIP in the synaptic cleft of trigeminal nerve and occipital nerve endings, affect intensity, duration and frequency of the headache attacks. This theory is bolstered by data showing a positive correlation of neurotransmitter concentrations in the synaptic cleft with amplitude and duration of neuronal activation.^294^ Taken together, it seems that data on molecular mechanisms can explain some of the similarities and differences in headache disorders, and that multiple complex molecular interactions rather than a single cascade of events is involved in the corresponding pathophysiologies.

#### Regional activity and connectivity pathways

Outcomes of regional activity and connectivity pathways show abnormal FA and FC in the pain matrix, DMN, salience and/or motor network, which are all important in perception, anticipation, processing and amplification of pain^295-297^ (Fig. 3, Tables 2–4 and Supplementary Tables 1 and 2). Notably, in all primary headache disorders, abnormalities are reported within different brainstem areas, suggesting heterogeneity in function (Tables 2–4 and Supplementary Tables 1 and 2). The brainstem appears to have variable involvement in the pathophysiology of primary headache disorders, including switching from interictal to ictal phase in migraine,^217^ fight-or-flight behavioural pattern in CH,^267^ as well as autonomic symptoms and chronification in PH and HC.^278^ Interestingly, ipsilateral brainstem activation from the attack site is reported for migraine and CH, whereas contralateral brainstem activation is observed for PH and HC.^298^ Though differences in brainstem activation could in part be explained by variability in the exact location and intensity of pain in the included subjects, this may also suggest differential connections to the trigeminovasular pathway in each side of the brainstem.^280,298^ For migraine, it is reported that following injection of sumatriptan, only abnormal brain stem activation persists, supporting the hypothesis that the pathogenesis is related to an imbalance between various brain stem nuclei involved in regulating antinociception and vascular control.^37^ An important structure closely connected to and located just above the brainstem, the hypothalamus, is thought to be a potential trigger for spontaneous migraine attacks,^299^ and a key factor in rhythmicity and autonomic symptoms in CH.^269,270^ Despite more recent evidence supporting the latter theories,^120,299,300^ initial functional imaging data showed no activity of hypothalamic activity in migraine and/or brainstem activity in CH during acute attacks.^37^ This difference may be explained by advancements in neuroimaging techniques, allowing for more accurate assessments of brain activity. When comparing functionality of the visual cortex, it appears to play a more central role in the pathophysiology of migraine, as only limited evidence of abnormalities within this area is reported for CH, PH, HC and ON (Tables 2–4 and Supplementary Tables 1 and 2). Though this may in part be due to lack of clinical investigation of this area in the other headache disorders, it supports the hypothesis that the visual cortex is involved in aura-like symptoms, which seem to be unique to migraine. With regard to the PFC, abnormalities are seen in migraine, CH, PH and HC, they do however differ from one another (Fig. 3). Increased FC of the PFC is present out of migraine attacks^205,226^ and during acute attacks of CH^262^ and PH and HC^279^ (Tables 2–4). This may suggest that abnormal emotional and cognitive processing of nociceptive signals by the PFC contributes to headache in CH, PH and HC, whereas in migraine, other areas are involved in amplification of pain during attacks. Notably, it is reported that PFC activation is less robust in PH and HC, with Evers and colleagues^274^ concluding that cognitive processing is involved in the pathophysiology of CH but not so much in PH. However, it is of note that since functional imaging data is rather limited for PH and HC, it remains unsure if altered PFC activity is either pivotal or an epiphenomenon. Taken together, neuroimaging studies show overlapping regional activity patterns and connectivity pathways in these headache disorders, however also indicate findings that seem specific to each diagnosis.

### Clinical implementation

**Figure 4:**
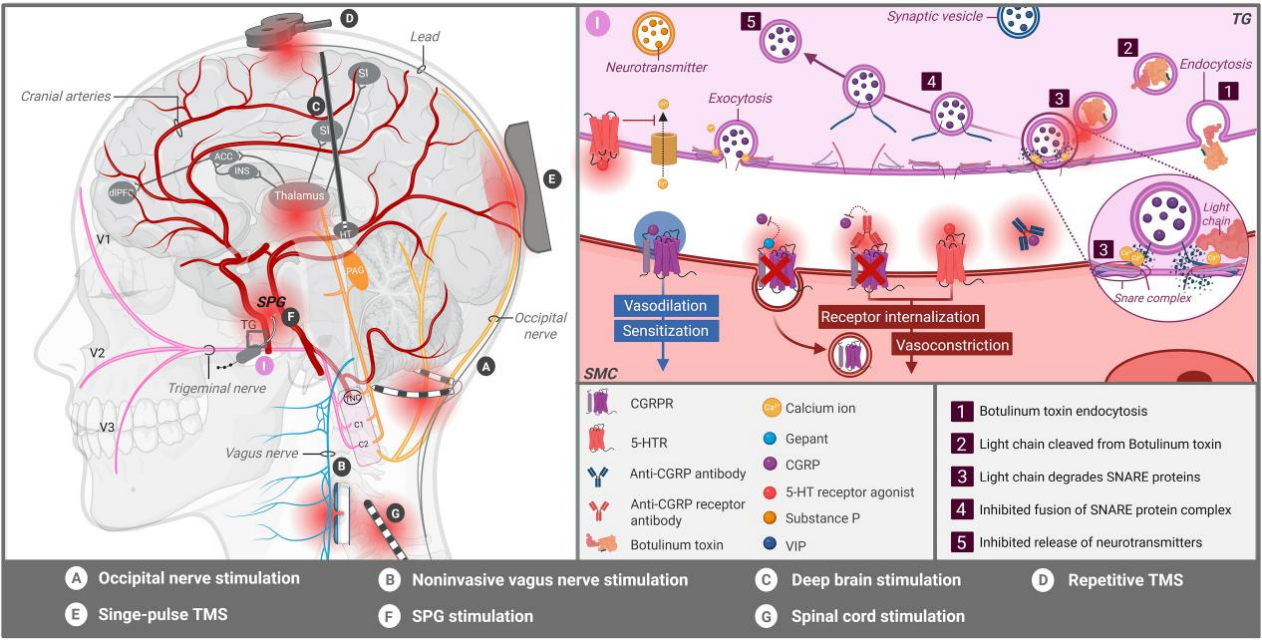
**Therapeutic options for headache disorders.** Mechanisms of therapeutic options for headache disorders, including non-invasive treatments [gepants, anti-CGRP (receptor) antibodies, triptans, non-invasive vagus nerve stimulation], minimally invasive options (botulinum toxin injectables), and neurosurgical alternatives (occipital nerve stimulation and deep brain stimulation of the hypothalamus). CGRP binds to CGRPR, leading to vasodilation and sensitization, contributing to headache pain. Gepants and anti-CGRP antibodies block CGRPR binding, causing vasoconstriction and eventually receptor internalization. Triptans activate 5-HT receptors, inducing vasoconstriction and inhibiting neurotransmitter release by preventing calcium influx, which is necessary for exocytosis of neurotransmitters in the trigeminal ganglion. Botulinum toxin is endocytosed into the trigeminal ganglion and degrades SNARE proteins, a complex necessary for fusion of membranes and release of neurotransmitters. Occipital nerve stimulation (**A**) modulates activity of the occipital nerve, while non-invasive vagus nerve stimulation (**B**) involves electrical stimulation of the vagus nerve. In deep brain stimulation (**C**) for headache disorders, electrical pulses are applied to the hypothalamus, thereby interfering with activity of brain regions involved in pain processing. Repetitive (**D**) and single-pulse (**E**) transcranial magnetic stimulation (TMS) are thought to improve headaches through modulation of the sensory trigeminal inputs at the thalamic level, while sphenopalatine ganglion (SPG) stimulation (**F**) interferes with the trigeminal autonomic pain reflex via the SPG. Spinal cord stimulation (**G**) is hypothesized to modulate activity of the trigeminal nucleus caudalis, which plays an important role in headache pathology. *Top right*: Representation of magnified region near the trigeminal ganglion, which is the primary site where most of these treatments exert their effects. ACC = anterior cingulate cortex; CGRP = calcitonin gene-related peptide; CGRPR = calcitonin gene-related peptide receptor; dlPFC = dorsolateral prefrontal cortex; 5-HT = serotonin; 5-HTR = serotonin receptor; INS = insula; PAG = periaqueductal grey; SMC = smooth muscle cell; SNARE = Soluble *N*-ethylmaleimide-sensitive factor activating protein receptor; TG = trigeminal ganglion; VIP = vasoactive intestinal peptide. Created in BioRender. Kollenburg, L. (2025) https://BioRender.com/xjwlef1.

Data on molecular pathways, regional activity patterns and connectivity pathways provide fundamental insights for clinical practice, leading to the emergence of new diagnostics and therapies.^66^ Throughout the years, these renewing insights led to the development of various approaches ranging from non-invasive treatments like gepants (e.g. Ubrogepant, Rimegepant and Zavegepant), CGRP monoclonal antibodies (e.g. Eptinezumab, Erenumab, Fremanezumab and Galcanezumab), triptans, nVNS and transcranial magnetic stimulation (TMS), to minimally invasive options like botulinum toxin injections, and more invasive alternatives like ONS, DBS, SPG stimulation and spinal cord stimulation (SCS)^7,301,302^ (Fig. 4). Ubrogepant, Rimegepant, and Zavegepant are small-molecule CGRP-receptor antagonists, Erenumab is a monoclonal antibody against the CGRP-receptor, and Eptinezumab, Fremanezumab, and Galcanezumab are monoclonal antibodies against the CGRP ligand.^303-305^ As a result, these treatments impair CGRP-induced nociceptive pathways (Fig. 4I). Triptans are selective 5-HT1b,d receptor agonists, acting on blood vessels and trigeminal nerve, preventing the release of neuropeptides^306^ (Fig. 4I). Botulinum toxin injections cause multiple cascade of events, with inhibiting the release of CGRP, glutamate and substance P.^307^ Botulinum toxin impairs the function of soluble *N*-ethylmaleimide-sensitive fusion-attachment protein receptor (SNARE) complex, a structure essential in fusion of synaptic vesicles to the membrane.^307^ As a result, neurotransmitter release in the trigeminal nerve is constrained^307^ (Fig. 4I). In contrast to previously mentioned targeted pharmacological treatments, ONS, nVNS, DBS, TMS, SPG stimulation and SCS do not act upon molecules directly but rather target the activity of specific nerves via the release of electrical pulses.^8,308^ In ONS, electrodes are positioned on the posterior aspect of the head, targeting the occipital nerves. It is theorized that ONS causes diffuse noxious inhibitory control (DNIC) via the TNC, thereby preventing activation of the secondary order neuron and spinothalamic tract^7,8^ (Fig. 4A). Non-invasive VNS modulates the activity of the vagus nerve and thereby interferes with the areas of the pain matrix trough its connections the trigeminal nerve and trigeminal spinal tract^309^ (Fig. 4B). In DBS for headache disorders, electrodes are often placed in the hypothalamus due to its central role in the pathology and connectivity to various areas of the pain matrix. As a result, this will lead to reduced activation of areas of the pain matrix, usually activated via the spinothalamic tract^310^ (Fig. 4C). TMS is a non-invasive neuromodulation technique that applies magnetic stimulation through a coil placed on the scalp. There are different forms of TMS available: repetitive TMS (rTMS), which delivers repeated pulses of magnetic stimulation, and single-pulse TMS (sTMS), which delivers individual magnetic pulses. TMS is believed to modulate sensory trigeminal inputs at the thalamic level, thereby reducing hyperactivity within the trigeminal system and alleviating headache symptoms^311^ (Figs. 4D and E). In SPG stimulation, electrodes are implanted through the infrazygomatic or transnasal route adjacent or directly on the SPG. Consequently, electrical stimulation will be applied to the SPG, thereby interfering with the trigeminal-autonomic pain reflex, which plays a major role in headache pathology^312^ (Fig. 4F). Finally, in SCS for headache disorders, electrodes are typically placed in the cervical region of the spinal cord. It is theorized that the electrical pulses delivered by SCS interfere with the altered activity of the TNC, thereby exerting an inhibitory effect on headache pain via modulation of the spinothalamic tract^313^ (Fig. 4G).

When comparing these treatment modalities, it becomes evident that each approach possesses a distinct mechanism of action. It is important to note that these therapies interfere with neuropeptides that serve a wide range of functions, extending beyond the pathways involved in the pathophysiology of headache disorders. Added to that, the pathophysiology of headache disorder is multifactorial; hence, care should be taken when considering these treatments. Taken together, advancing knowledge of molecular mechanisms, regional activity patterns and connectivity pathways, alongside emerging evidence supporting the potential of existing targeted therapies, fuels a growing interest in the development of prospective targeted therapies for headache disorders among different specialities, including neurology, anaesthesiology and neurosurgery. Prospective studies are currently focusing on finding new potential targets for headache disorders, like VIP^48^ and PACAP.^314,315^

### Strengths and limitations

Limitations of this literature review include the wide publication time span (1976–2024), the heterogeneity of study populations across the included articles, and the considerable variability in the volume of available literature between different headache disorders. Headache disorders consist of several subtypes, for instance, migraine with and without aura, which cannot be fully corrected for, as these subtypes are often analysed as a single group.^184,192,209^ On this matter, it is important to note that the pathophysiological theories provided in this article may vary among subtypes. Another factor that complicates the current analysis is the inclusion of mixed study populations in several articles, particularly concerning HC and PH, despite these being clinically distinct entities. We were aware of this issue prior to conducting the analysis. However, it was chosen to present HC and PH within the same section to allow for the inclusion of studies involving mixed cohorts. Given the limited availability of data, overlapping pathophysiological features, and the presence of studies with combined populations, it has been decided that retaining these studies in the analysis and grouping the outcomes under a shared section was the most practical approach, while clearly maintaining that HC and PH are separate diagnoses. Another restriction of the current analysis is the fact that multiple headache disorders are often present simultaneously as co-morbidities in certain patients, hence making it difficult to investigate headache disorders separately from one another. Though noted in Tables 2–4, we did not separately discuss outcomes for triggered versus spontaneous headache attacks, as this would compromise the clarity and comprehensibility of the article. Additionally, advancements in neuroimaging and molecular analyses are also likely to have affected current outcomes. Furthermore, a subset of studies in this analysis includes data from non-human species^50,62,87,136,292^; hence, caution is warranted when interpreting these reports. We were aware of these restrictions prior to the analysis; however, due to the limited publication of articles on PH, HC and ON, exclusion criteria were not adjusted. Nevertheless, we considered it important to include PH, HC and ON despite the limited data, as identification of this scarcity itself is relevant, highlighting existing gaps in the literature and underscoring the need for future research to better understand the underlying mechanisms involved.

## Conclusion

Headache disorders are complex neurological disorders, owing to the involvement of a variety of molecular mechanisms, regional activity patterns and connectivity pathways in their pathophysiology. Combining molecular, regional activity and connectivity data improves the understanding of these disorders and provides fundamental insights for developing innovative treatment strategies. Prospective studies should focus on further exploring potential targets in these pathways, broadening the treatment spectrum for headache disorders.

## Supplementary Material

awaf361_Supplementary_Data
