# Supplementary material for: Bridging the gap: molecular mechanisms, regional activity and connectivity in headache disorders
Source: Brain. 2025 Sep 25;149(3):710–35. doi: 10.1093/brain/awaf361 (PMC13017448; doi:10.1093/brain/awaf361)
Supplement: awaf361_Supplementary_Data [file awaf361_supplementary_data.pdf]

Supplementary Table 1: Overview of functional and structural changes in migraine

Author (Year)	*Study design[1]*	*Number of patients*	*diagnosis*	*Experimental paradigm*	*Phase of measurement*	*Imaging modality*	*Results*
Weiller et al (1995)[1]	I, LS	N= 9 (M)	MwoA	Injection of 6mg sumatriptan	During attack, headache free intervals	PET	**During attack vs attack free intervals**: higher CBF in cerebrum, bilaterally in cingulate cortex, inferior anterocaudal cingulate cortex, auditory association cortices and parieto-occipital junction in the visual association cortex and brainstem<br>**Sumatriptan:** no increase in CBF in cortical areas of hemispheres compared to headache free-interval
Hadjikhani et al (2001)[2]	I, LS, CC	N=3 (M), N=7 (HCo)	MwA	Triggered attacks with exercise	Ictal and interictal phase	fMRI	**MwA versus HCo**: no differences in pattern of activation in visual cortex.<br>**Induced attacks in ictal versus interictal phase of MwA:** increased FA in visual cortex
Mainero et al (2011)[3]	O, CC	N=17 (M), N=17 (HCo)	MwA(N=8) and MwoA(N=9)	N/A	Interictal	rs-fMRI	**MwA/MwoA versus HCo:** increased FC of PAG with right ventrolateral PFC, right supramarginal gyrus, right anterior insula, right thalamus, left angular gyrus, left supramarginal gyrus/parietal operculum, bilateral precentral gyrus and right postcentral gyrus. Decreased FC with left medial PFC, left anterior insula, left dorsomedial PFC, right lateral PFC and right ACC
Russo et al (2012)[4]	O, CC	N=14 (M), N=14 (HCo)	MwoA	N/A	Interictal period	rs-fMRI	**MwoA versus HCo:** decreased FC with right middle frontal gyrus and right dorsal ACC. Reduced FC with the middle frontal gyrus is negatively correlated with pain intensity of migraine attacks
Xue et al (2012)[5]	O, CC	N=23 (M), N=23 (HCo)	MwoA	N/A	N/A	rs-fMRI	**MwoA versus HCo:** for the CEN, there is increased FC with right middle frontal gyrus, right anterior insula and left inferior frontal gyrus. In the SN, decreased FC with right SMA, and for the DMN, increased FC with right anterior insula is found
Yu et al (2012)[6]	O, CC	N=26 (M), N=26 (HCo)	MwoA	N/A	Interictal phase	rs-fMRI	**MwoA versus HCo:** reduced regional homogeneity in the rostral ACC, PFC, OFC and SMA. Regional homogeneity in the rostral ACC and PFC is negatively correlated with duration of disease
Yuan et al (2012)[7]	O, CC	N=21 (M), N=21 (HCo)	MwoA	N/A	Not during attack	DTI and rs-fMRI	**MwoA versus HCo:** increased FC of the right ACC with bilateral OFC, and left ACC with bilateral OFC and right dlPFC
Hadjikhani et al (2013)[8]	O, CC	N=22 (M), N=20 (HCo)	MwA(N=11) and MwoA(N=11)	N/A	Interictal	rs-fMRI	**MwA/MwoA versus HCo:** increased FC from amygdala with anterior insula, S2 and thalamus
Jin et al (2013)[9]	O, CC	N=21 (M), N=21 (HCo)	MwoA	N/A	Not during attack	rs-fMRI	**MwoA versus HCo:** increased FC of dorsal ACC with bilateral middle temporal lobe, OFC and left dorsolateral PFC. Increased FC of right cerebellum with right medial PFC, and left medial PFC with bilateral dorsolateral PFC. Increased FC of right occipital lobe with left dorsolateral PFC and right MCC. No changes in brainstem FC
Schwedt et al (2013)[10]	O, CC	N=20 (M), N=20 (HCo)	CM	N/A	Interictal phase	rs-fMRI	**CM versus HCo:** atypical FC of amygdala with superior frontal cortex and occipital cortex, and anterior insula with precuneus, PAG, mediodorsal thalamus, middle temporal cortex, inferior parietal cortex and pulvinar
Tessitore et al (2013)[11]	O, CC	N=20 (M), N=20 (HCo)	MwoA	N/A	Interictal phase	rs-fMRI	**MwoA versus HCo:** in the DMN, decreased FC with left superior prefrontal gyrus and left temporal pole
Xue et al (2013)[12]	O, CC	N=18 (M), N=18 (HCo)	MwoA	N/A	Interictal phase	rs-fMRI	**MwoA versus HCo:** increased FC of left PFC with precuneus and bilateral parietal lobe and left ACC with frontal and parietal lobe. Increased FC of right PFC with bilateral parietal lobe and left temporal lobe and right insula with left temporal pole, right frontal lobe and left parietal lobe
Yuan et al (2013)[13]	O,CC	N=40 (M), N=40 (HCo)	MwoA	N/A	Interictal phase	sMRI and rs-fMRI	**MwoA versus HCo**: increased FC of left caudate t bilateral hippocampal gyrus, left amygdala, left putamen and left insula, and of right nucleaus accumbens with bilateral parrahippocampal gyrus, bilateral ACC, left PCC and bilateral OFC. Increased FC of right caudate with left insula and left putamen

Maniyar et al (2014)[14]	I, LS	N=8 (M)	MwoA	Triggered attacks with Nitroglycerin	Preictal phase and during attack	PET	**Preictal phase at baseline versus triggered attack in MwoA:** increased FA of posterolateral hypothalamus, midbrain tegmental area, PAG, dorsal pons and various cortical areas including PFC, occipital and temporal cortex during triggered attack
Moulton et al (2014)[15]	O, CC	N=12 (M), N=12 (HCo)	MwoA	N/A	Interictal phase	fMRI	**MwoA versus HCo:** increased FC of hypothalamus with right precentral gyrus, right middle frontal gyrus and eft superior parietal gyrus, right planum polare, left inferior temporal gyrus, bilateral hippocampus, left caudate right nucleus coeruleus, bilateral pontine nuclei, bilateral cerebellum and left dentalte nucleus. Decerased FC of precentral gyrus, left paracingulate gyrus, left superior frontal gyrus, left frontal pole, right fusiform gyrus and left lingual gyrus
Hougaard et al (2015)[16]	O, CC	N=40 (M), N=40 (HCo)	MwA	N/A	Interictal	rs-fMRI	**MwA versus HCo:** no changes in FC of cortical visual areas, amygdala and PAG
Li et al (2015)[17]	I, CC	N=12 (M), N=12 (HCo)	MwoA	4 weeks standard acupuncture treatment	Not during attacks	rs-fMRI	**MwoA versus HCo:** decreased FC in the right frontoparietal network which could be reversed by acupuncture treatment. Changes in FC are negatively correlated with decease in visual analogue scale after treatment
Amin et al (2016)[18]	RCT	N=24 (M)	MwoA	IV administration of PACAP38 (N=16) or VIP (N=15) over 20 minutes	During attack	rs-fMRI	**PACAP38:** increased FC with bilateral opercular part of inferior frontal gyrus in the SN, right PMC and decreased FC with left visual cortex. For the DMN, increased FC in left primary auditory cortex, S2, PMC and visual cortex, and decreased FC in the right cerebellum and left frontal lobe.<br>**VIP**: no resting-state network changes
Coppola et al (2016)[19]	O, CC	N=13 (M), N=19 (HCo)	EMwoA	N/A	During attack	rs-fMRI and DTI	**EMwoA vs HCo:** decreased FC between DAS and ECN, no differences in thalamic FA
Hodkinson et al (2016)[20]	O, LS, CC	N=40 (M), N=40 (HCo)	EM	N/A	Before, during and after attack	rs-fMRI	**EM vs HCo:** decreased FC to areas of the inferior/middle occipital cortex and precuneus. In auditory network, reduced FC to regions of PFC (dlPFC, PCC and lateral parietal cortex), insula, operculum and anterior temporal lobe. No significant changes in somatosensory network. Reduced FC with midline medial prefrontal and parietal areas (MCC, PCC, mPFC/dlPFC)
Niddam et al (2016)[21]	O, CC	N=52(M), N=26 (HCo)	Mwo (N=26) and MwoA (N=26)	N/A	N/A	rs-fMRI	**MwA versus HCo**: in DAS, increased FC with right orbital gyrus, left rectal gyrus, right fusiform gyrus, right middle temporal gyrus and right parrahippocampal gyrus. In SN, decreased FC with bilateral cuneus, left superior occipital gyrus, right lingual gyrus, left fusiform gyrus and left middle temporal gyrus. For the right cuneus, increased FC with left cingulate gyrus, bilateral precuneus and bilateral posterior cingulate. Decreased FC with bilateral insula, bilateral middle frontal gyrus and bilateral cingulate gyrus and bilateral superior frontal gyrus<br>**MwoA versus HCo**: increased FC with middle temporal gyrus, right parahippocampal gyrus, right middle occipital gyrus and right fusiform gyrus<br>**MwA versus MwoA**: in the SN, decreased FC with the bilateral cuneus, bilateral lingual gyrus, left middle temporal gyrus and middle occipital gyrus. Increased FC with the left middle frontal gyrus, bilateral cingulate gyrus and right precuneus. Decreased FC with bilateral insula, bilateral claustrum, right lentiform nucleus, right inferior frontal gyrus and left middle frontal gyrus
Schulte and May (2016)[22]	O, CR	N=1 (M)	EMwoA	N/A	Ictal and interictal phase	fMRI	**Baseline versus attack in EMwoA:** increased FA of pons and decreased FA of visual cortex. Increased FC between the pons and hypothalamus

Tedeschi et al (2016)[23]	O, CC	N=40 (M), N=20 (HCo)	MwA (N=20) and MwoA (N=20)	N/A	Interictal period	rs-fMRI	**MwA versus MwoA and HCo:** increased FC with right lingual gyrus
Zhang et al (2016)[24]	O, CC	N=22 (M), N=22 (HCo)	MwoA	N/A	Not during attacks	fMRI	**MwoA versus HCo:** within the DMN, increased FC of the visuospatial system and medial visual cortical areas
Androulakis et al (2017)[25]	O, CC	N=29 (M), N=29 (HCo)	CM	N/A	N/A	rs-fMRI	**CM versus HCo:** decreased FC of DMN, SN and CEN
Arngrim et al (2017)[26]	I, CS	N=5 (M)	EMwA	Hypoxia triggered attacks (N=3), sham hypoxia (N=1), triggered attacks by exercise and photostimulation (N=1)	During attack	fMRI	**Baseline versus EMwA:** altered activation of the of visual cortex
Chen et al (2017)[27]	O, CC	N=34 (M), N=18 (HCo)	CM (N=16) and EM (N=18)	N/A	Interictal phase	fMRI	**EM versus HCo:** increased FC in left amygdala with left middle cingulate gyrus and left precuneus and no change in right amygdala<br>**CM versus HCo:** no change in left amygdala and decreased FC from right amygdala to right inferior occipital lobe and right middle occipital lobe<br>**CM vs EM:** increased FC of bilateral amygdala
Hougaard et al (2017)[28]	O, LS	N=16 (M)	MwA	N/A	During attacks, headache free intervals	rs-fMRI	**During attacks versus headache free intervals in MwA:** increased FC during attacks between the left pons and left S1 including head and face somatropic areas, increased FC during attacks between visual area and lower middle frontal gyrus in symptomatic hemisphere
Lo Buono et al (2017)[29]	O, CC	N=28 (M), N=14 (HCo)	MwA (N=14) and MwoA (N=14)	N/A	Interictal phase	fMRI	**MwoA versus HCo**: in the DMN, increased FC to the occipital pole, cingulate gyrus, occipital fusiform gyrus and bilateral lingual gyrus<br>**MwA versus HCo**: increased FC to the bilateral planum temporale, left superior temporal gyrus and bilateral Heschl's gyrus<br>**MwA versus MwoA:** in the DMN, increased FC to the Heschl's gyrus, left superior temporal gyrus, bilateral lingual gyrus, right occipital fusiform gyrus, right insular cortex, left occipital pole and central opercular cortex
Schulte et al (2017)[30]	O, CC	N=35 (M), N=19 (HCo)	EM (N=18) and CM (N=17)	N/A	Ictal phase during and not during attacks	fMRI	**EM/CM with headache versus EM/CM without headache and HCo:** increased FA of posterior hypothalamus

Yu et al (2017)[31]	O, CC	N=31 (M), N=31 (HCo)	MwoA	N/A	Not during attack	fMRI	**MwoA versus HCo:** decreased FC of the left PFC with insula and posterior parietal cortex, and of right ACC with PFC and PCC
Zhang et al (2017)[32]	O, CC	N=30 (M), N=31 (HCo)	MwoA	N/A	Headache free periods	rs-fMRI	**MwoA versus HCo:** increased FC of left S1 with the anterior parietal lobe, right superior parietal lobe, right S1, bilateral PMC, right inferior frontal gyrus, right insula, right temporal lobe, left M1 and right middle occipital gyrus. Decreased FC of the right S1 with the left inferior parietal lobule, right cerebellum lobule, right tempotal lobe, bilateral paracentral lobule, bilateral S1, bilateral ACC, pons, left insula. Bilateral frontal gyrus and bilateral PMC
Amin et al (2018)[33]	O, LS	N=17 (M)	EMwoA	N/A	Ictal and interictal phase	rs-fMRI	**Baseline versus attacks IN EMwoA:** increased FC of thalamus with M1 and OFC, parietal brain areas, insula, SMA and decreased thalamic FC with S1 and PMC
Meylakh et al (2018)[34]	O, LS, CC	N=26 (M), N=78 (HCo)	M	N/A	Before, after and between migraine attacks	fMRI	**M versus HCo:** preceding an attack and during attack, increased FA in brainstem, and hypothalamic regions (spinal trigeminal nucleus, dorsal pons, hypothalamus) is present, and remained unchanged directly after or between attacks and were significantly different from HCo
Coppola et al (2019)[35]	O, CC	N=20 (M), N=20 (HCo)	CM	N/A	N/A	MRI	**CM versus HCo:** reduced FC between DMN and ECN. In DAS, increased FC with DMN and weaker with ECN
Lee et al (2019)[36]	O, CC	N=62 (M)	CM (N=18) and EM (N=44)	N/A	Interictal	rs-fMRI	**CM versus EM:** increased connectivity in pain matrix (dlPFC, anterior insula, ACC, thalamus and precuneus
Russo et al (2019)[37]	I, CC	N=17 (M), N=15 (HCo)	MwA	Trigeminal noxious heat stimulation	Interictal phase	fMRI	**MwA versus HCo:** increased FA in lingual gyrus, inferior parietal lobule, inferior frontal gyrus and medial frontal cortex, cerebellum
Coppola et al (2020)[38]	O, CC	N=20 (M), N=20 (HCo)	CM	N/A	During attack	rs-fMRI	**CM vs HCo:** increased FC between from hypothalamus to mPFC, left dorsal visual network and bilateral lobules. No alterations between hypothalamus and brainstem.
Karsan et al (2020)[39]	I, LS	N=21 (M)	MwA (N=11) and MwoA (N=10)	Triggered attacks with Nitroglycerin (N=21) and placebo administration (N=21)	During headache and preictal phase	rs-fMRI	**Baseline versus triggered attack in MwA/MwoA:** increased FC of the pons with the cerebellar tonsils, medulla and limbic cortical areas. Decreased FC between pons and limbic cortical areas **Basleine versus triggered attacks and placebo versus nitroglycerin in MwA/MwoA:** decreased thalamic connectivity with cuneus and precuneus in nitroglyceirn group

Schulte et al (2020)[40]	O, LS	N=8 (M)	EMwA (N=1) and EMwoA (N=7)	N/A	Ictal, interictal and preictal phase	rs-fMRI	**Baseline versus attacks in EMwA/EMwoA:** increased FC between pons with hypothalamus and nucleus accumbens
Veréb et al (2020)[41]	O, CC	N=57 (M) N=32 (HCo)	MwA (N=20) and MwoA (N=37)	N/A	Interictal period	rs-fMRI	**MwA versus MwoA:** increased FC between right insula and dorsal ACC<br>**MwA versus HCo:** increased FC between dorsal ACC and left PFC<br>**MwoA versus MwA and HCo**: decreased FC between right insula and PFC
Maleki et al (2021)[42]	I, LS	N=19 (M)	EMwA and EMwoA	Painful heat stimulation of the hand	Ictal and interictal phase	fMRI	**Noxious stimulation in no allodynia vs generalized allodynia in EMwA/EMwoA**: increased trigeminal nucleus and thalamus, but no differences in FA trigeminal ganglion<br>**Ictal versus interictal phase in EMwA/EMwoA:** alterations in brainstem/pons, thalamus, insula, cerebellum and cingulate cortex
Martinelli et al (2021)[43]	I, LS	N=5 (M)	EMwoA	Triggered attacks with Nitroglycerin	Pain-free condition, prodromal and full-blown phase of attack	rs-fMRI	**Baseline versus attack in EMwoA:** decreased FC thalamus and pons, orbital gyrus, cerebellum
Van Oosterhout et al (2021)[44]	I, LS, CC	N=12 (M), N=10 (HCo)	EMwoA	Attacks triggered with nitroglycerin (N=12)	Preictal and interictal phase	fMRI	**Baseline versus triggered or spontaneous attacks in EMwoA:** increase in hypothalamic activity in triggered and spontaneous attacks<br>**Baseline versus attacks in HCo:** no functional changes
Cao et al (2022)[45]	O, LS, CC	N=44 (M), N=44 (HCo)	MwoA	N/A	Interictal phase, during an attack	rs-fMRI	**MwoA versus HCo:** decreased grey matter in middle frontal gyrus<br>**MwoA with headache versus HCo:** increased FC between middle frontal gyrus and cerebellum<br>**MwoA interictal phase versus HCo:** decreased FC in cingulum, superior frontal gyrus and middle frontal gyrus and precuneus<br>**Interictal versus during attacks in MwoA:** decreased FC between middle frontal gyrus and superior frontal gyrus
Gollion et al (2022)[46]	O, CC	N=21 (M), N=18 (HCo)	MwA	N/A	Interictal	rs-fMRI	**MwA versus HCo:** enhanced FC of the right and left anterodorsal insula and clusters in the upper cerebellum
Porcaro et al (2022)[47]	O, CC	N=15 (M), N=20 (HCo)	EMwoA	N/A	Ictal phase	DTI and rs-fMRI	**EMwoA versus HCo:** decreased fractional anisotropy of hypothalamus
Jang et al (2023)[48]	O, CS	N=27 (M)	MwoA	N/A	No migraine attack (interictal stage)	MRI	**Out of attack in MwoA:** ipsilateral versus contralateral hemispheres in MwoA: No significant differences in CBF

ACC, anterior cingulate cortex; CBF, cerebral blood flow; CC, case control study; CEN, central executive network; CM, chronic migraine; CR, case report; CS, case series; DAS, dorsal attention system; dlPFC, dorsolateral prefrontal cortex; DMN, default mode network; DTI, diffusion tensor imaging; EM, episodic migraine; FA, functional activity; FC, functional connectivity; fMRI, functional magnetic resonance imaging; HCo, healthy controls; I, interventional study; LS, longitudinal study; M, migraine; M1, primary motor cortex; MCC, medial cingulate cortex; mPFC, medial prefrontal cortex; MRI, magnetic resonance imaging; N/A, not available or not applicable; O, observational study; OFC, orbitofrontal cortex; PAG, periaqueductal gray; PCC, posterior cingulate cortex; PET, positron emission therapy; PFC, prefrontal cortex; PMC, premotor cortex; RCT, randomized controlled trial; rs-fMRI, resting state magnetic resonance imaging; S1, primary somatosensory cortex; S2, secondary somatosensory cortex; SMA, supplementary motor area; sMRI, structural magnetic resonance imaging; SN, salience network; wA, with aura; WoA, without aura. [1]Please note that other review articles are not included within this table.

Supplementary Table 2: Overview of functional and structural changes in cluster headache

Author (Year)	*Study design[1]*	*Number of patients*	*diagnosis*	*Experimental paradigm*	*Phase of measurement*	*Imaging modality*	*Results*
Henry et al (1978)[49]	O, CS	N=3 (CH)	ECH	N/A	During attacks	SPECT	**During attacks of ECH:** no alterations in CBF
Sakai et al (1978)[50]	O, LS	N=71 (M, CH, TTH) N=32 HCo)	M (N=43), CH (N=9), TTH (N=19)	N/A	During and out of attacks	SPECT	**During versus out of CH attack:** significant increase in CBF
Krabbe et al (1984)[51]	I, LS	N=18 (CH)	CCH (N=9), ECH (N=9)	Triggered attacks with NTG and/or alsochol	During (N=8) and out of attacks	SPECT	**Baseline versus attack in CCH and ECH:** increased rCBF in basal region, central and parieto-temporal region. No significant changes in CBF
Hsieh et al (1996)[52]	I, LS	N=7 (CH)	ECH	Triggered attacks with NTG	In-bout (N=4), out-of-bout (N=3)	PET	**In versus out-of bout:** decreased rCBF in PFC, posterior parietal and occipital-temporal cortex. Increased rCBF in right and rostrocaudal ACC, temporo-polar cortex, SMA, insula, putamen, lateral inferior frontal cortex, motor cortex and PMC and lateral inferior frontal cortex
Di Piero et al (1997)[53]	O, CC	N=7 (CH), N=12 (HCo)	ECH	N/A	Out-of-bout	SPECT	**ECH versus HCo:** decreased CBF in contralateral M1 and contralateral thalamus
May et al (1998)[54]	I, LS	N=17 (CH)	CCH (N=9), CH (N=8)	Triggered attacks with NTG	During attack (N=9) and out-of bout (N=9)	PET	**During attack versus out-of bout CCH:** increased FC in ipsilateral hypothalamic grey area, contralateral ventroposterior thalamus, ACC and bilaterally in insula during attack. Hypothalamic activation was not seen in those being out-of-bout
May et al (1999)[55]	O, LS, CC	N=25 (CH), N=29 (HCo)	ECH and CCH	N/A	In-bout and out-of-bout	VBM	**CH versus HCo:** increased density in posterior hypothalamus grey matter. **In-bout versus out-of-bout:** no differences
May et al (2000)[56]	I, LS, CC	N=17 (CH), N=4 (HCo)	CCH (N=9), ECH (N=8)	**MRA study:** pain in HCo is induced with capsaicin (N=4) **PET study:** NTG is used to induce CH attacks (N=9, induced; N=1 spontanious)	Out-of-bout (N=8) and in-bout (N=9)	PET and MRA	**Induced pain in HCo versus spontanious CH attack:** both groups show Increased CBF in internal carotid artery (ipsilateral from headache site) **In versus out-of-bout of CH attacks:** increased FA in ACC, ipsilateral posterior hypothalamus, contraletaral ventroposterior hypothalamus, ipsilateral basal ganglia, frontal lobes, blateral insula and contralateral inferior frontal cortex
Sprenger et al (2004)[57]	I, CR	N=1 (CH)	CCH	Attacks terminated with sumatriptan	Out of and during attack	PET	**Baseline versus attack CCH:** increased FA of contralateral perigenual ACC, ipsilateral hypothalamus and medial thalamus
Lodi et al (2006)[58]	O, LS, CC	N=26 (CH), N=12 (HCo)	ECH (N=18), CCH (N=8)	N/A	In-bout and out-of bout	H-MRS	**CH versus HCo:** reduced NAA/Cr and NAA/cho and no significant changes in Cr **In- and out-of-bout ECH versus CCH:** reduced hypothalamic NAA/Cr in and out-of-bout in ECH and CCH
Wang et al (2006)[59]	O, LS, CC	N=63 (CH, M) N=21 (HCo)	ECH (N=47), CM (N=16)	N/A	In-bout (N=35) and out-of-bout (N=12)	H-MRS	**CH versus HCo and migraine:** decreased NAA/Cr and Cho/Cr in hypothalamus. **In-and-out of bout:** no significant differences in NAA/Cr and Cho/Cr
Sprenger et al (2007)[60]	O, LS, CC	N=11 (CH), N=11 (HCo)	ECH	N/A	In-bout and out-of bout	FDG-PET	**In-bout versus out-of bout in CH:** increased metabolism in ACC, perigenual PCC, PFC, insula, thalamus and temporal cortex. Decreased metabolism in cerebellopontine area **CH versus HCo:** decreased metabolism in perigenual ACC, PFC and OFC
Morelli et al (2009)[61]	I, LS	N=4 (CH)	ECH	Attacks terminated with sumatriptan	During and after attack	fMRI	**During versus after attack:** increased FA in ipsilateral hypothalamus

Rocca et al (2010)[62]	O,CC	N=13 (CH), N=15 (HCo)	ECH	N/A	Out of headache	rs-fMRI	**ECH versus HCo:** increased FC with hypothalamus and thalamus. Decreased fluctuations in the sensimotor and visual betwork. Abnormalities in resting state network are related to disease duration
Qiu et al (2012)[63]	O,LS, CC	N=12 (CH), N=12 (HCo)	ECH	N/A	In-bout during and out of attacks	rs-fMRI	**During versus out of CH attack:** increased FC of hypothalamus with ACC, PCC, superior frontal gyrus, middle frontal gyrus, superior temporal gyrus, inferior frontal gyrus, inferior parietal lobule, amygdala and parahippocampal gyrus<br>**Out of attack CH versus HCo:** increased FC of hypothalamus with frontal, parietal and temporal cortex. No significant changes in FA of hypothalamus with ACC and PCC. Decreased FC between hypothalamus and occipital cortex
Steinberg et al (2012)[64]	O, LS, CC	N=14 (CH) N=5 (HCo)	ECH	N/A	In-bout and out-of-bout	WBC-SPECT	**ECH in-bout versus HCo and CH out-of-bout:** no significant alterations in white blood cell uptake
Morelli et al (2013)[65]	I, LS	N= 4 (CH)	ECH	Attacks terminated with sumatriptan	Before, during and after attack	fMRI	**During attack versus pain-free state:** increased FA of ipsilateral hypothalamus, ipsilateral trigeminal root entry zone, bilateral red nucleus and ventral pons without lateraliztion
Chou et al (2014)[66]	I, LS, CC	N=17 (CH), N=17 (HCo)	ECH	N/A	In-bout, out of attack	DTI	**ECH versus HCo:** higher absolute mean dffusity frontal region and lower absolute diffusities in limbic system. Increased FC between altered areas and hypothalamus<br>**In-bout versus out-of bout:** altered microstructure of left cerebellar tonsil
Qiu et al (2015)[67]	O, CC	N=21 (CH), N=21 (HCo)	ECH	N/A	In-bout	rs-fMRI	**ECH versus HCo:** decreased hypothalamic SN coactivation.
Yang et al (2015)[68]	O, LS, CC	N=18 (CH), N=19 (HCo)	ECH	N/A	In-bout and out-of-bout	rs-fMRI	**ECH versus HCo:** altered FC of hypothalamus with medial frontal gyrus and occipital cuneus<br>**In-bout versus out-of bout:** decreased FC of hypothalamus with medial frontal gyrus, precuneus and cerebellar areas (tonsil, declive and culmen)
Arkink et al (2017)[69]	O, CC	N=103 (CH, M, CPH), N=48 (HCo)	ECH (N=24), CCH (N=23), probable CH (N=14), MwA (N=14), MwoA (N=19), CPH (N=9)	N/A	N/A	VBM	**ECH/CCH versus HCo:** bilateral enlargement of the suprachiasmatic and paraventricular nuclei in ECH and CCH
Chou et al (2017)[70]	O, LS, CC	N=17 (CH), N=18 (HCo)	ECH	N/A	In-bout and out-of bout	rs-fMRI	**ECH versus HCo:** increased FC in temporal, frontal, SN, DMN, somatosensory, DAS and visual network independency from bout period.<br>**in-bout versus out-of-bout:** altered FC in the frontal and DAS
Faragó et al (2017)[71]	O, CC	N=17 (CH), N=17 (HCo)	ECH	N/A	Out-of-bout	rs-fMRI	**ECH versus HCo:** increased FA in ipsilateral attention network and contralateral cerebellar network.
Ferraro et al (2018)[72]	O, CC	N=17 (CH), N=16 (HCo)	CCH	N/A	In-bout	rs-fMRI	**CH versus HCo:** increased FC of ipsilateral posterior hypothalamus with VTA, dorsal raphe nuclei and bilateral substantia nigra, red nucleus and subthalamic nucleus. No differences in FC pf contralateral hypothalamus
Ha et al (2019)[73]	O, CC	N=10 (CH), N=20 (HCo)	ECH	N/A	N/A	MRI	**ECH versus HCo:** increased strength and closeness of cingulate gyrus. Decreased volume of caudal ACC and postcentral gyrus
Chong et al (2020)[74]	O, CC	N=37 (M, CH), N=22 (HCo)	ECH (N=18), M (N=19)	N/A	Out-of-bout	VBM	**CH versus HCo and migraine:** decreased FC of hypothalamus with frontal and temporal-parietal pain control system. No significant differences in hypothalamic region volume or total brain volume

Giorgio et al (2020)[75]	O	N=25 (CH, M)	ECH (N=12), MwoA (N=13)	N/A	Out of attack	rs-fMRI	**CH versus migraine:** increased FC within working memory and executive control networks
Möller et al (2020)[76]	I, LS	N=26 (HCo)	N/A	Autonomic symptoms provoked by administration of Kinetic Oscillation Stimulation in the nostral	N/A	fMRI	**Baseline versus during triggered autonomic symptoms:** induced activation of brainstem and cerebellar regions an bilateral insular regions after nonpainful stimuli and enhanced FA of locus coeruleus, ventral posteriomedial nucleus of thalamus, anterior hypothalamus and ipsilateral insula following painful stimuli
Ferraro et al (2022)[77]	I, CC	N=28 (CH), N=28 (HCo)	CCH	N/A	After CH attack	sMRI and rs-MRI	**sMRI CCH versus HCo:** Increased volume of bilateral nucleus accumbens, ventral diencephalon, hippocampus, frontal pole and right amygdala. Altered volume is present ipsilaterally to the pain ventral diencephalic regions and contralateral tot he pain nucleus accumbens<br>**fMRI CCH versus HCo:** reduced FC in right prontal pole-right amygdala pathway
Messina et al (2022)[78]	O, CC	N=40 (CH, M) N=15 (HCo)	ECH (N=20), M (N=20)	N/A	Out-of-bout, out of attacks	MRI	**CH and migraine versus HCo:** alterations in bilateral hypothalamic and PAG functional networks<br>**CH versus migraine**: decreased functional interaction with left thalamus and cortical areas mediating interoception and sensory integration

ACC, anterior cingulate cortex; CBF, cerebral blood flow; CC, case control study; CCH, chronic cluster headache; CH, cluster headache; CPH, chronic paroxysmal hemicrania; CR, case report; CS, case series; DAS, dorsal attention system; DMN, default mode network; DTI, diffusion tensor imaging; ECH, episodic cluster headache; FA, functional activity; FC, functional connectivity; FDG-PET, fluorodeoxyglucose; fMRI, functional magnetic resonance imaging; HCo, healthy controls; H-MRS, proton magnetic resonance spectroscopy; I, interventional study; LS, longitudinal study; M, migraine; M1, primary motor cortex; MRA, magnetic resonance angiography; MRI, magnetic resonance imaging; N/A, not available or not applicable; O, observational study; OFC, orbitofrontal cortex; PAG, periaqueductal gray; PCC, posterior cingulate cortex; PET, positron emission therapy; PFC, prefrontal cortex; PMC, premotor cortex; rCBF, regional cerebral blood flow; rs-fMRI, resting state magnetic resonance imaging; SMA, supplementary motor area; sMRI, structural magnetic resonance imaging; SN, salience network; SPECT, single positron emission tomography; TTH, tension type headache; VBM, voxel-based morphometry; WBC-SPECT, white blood cell single poistron emmision tomography. [1]Please note that other review articles are not included within this table.

References

1. C. Weiller, A. May, V. Limmroth, et al. Brain stem activation in spontaneous human migraine attacks. *Nat Med*. Jul 1995;1(7):658-660. doi:10.1038/nm0795-658
2. Nouchine Hadjikhani, Margarita Sanchez del Rio, Ona Wu, et al. Mechanisms of migraine aura revealed by functional MRI in human visual cortex. *Proceedings of the national academy of sciences*. 2001;98(8):4687-4692.
3. Caterina Mainero, Jasmine Boshyan, Nouchine Hadjikhani. Altered functional magnetic resonance imaging resting-state connectivity in periaqueductal gray networks in migraine. *Annals of neurology*. 2011;70(5):838-845.
4. Antonio Russo, Alessandro Tessitore, Alfonso Giordano, et al. Executive resting-state network connectivity in migraine without aura. *Cephalalgia*. 2012;32(14):1041-1048.
5. Ting Xue, Kai Yuan, Ling Zhao, et al. Intrinsic brain network abnormalities in migraines without aura revealed in resting-state fMRI. *PloS one*. 2012;7(12):e52927.
6. Dahua Yu, Kai Yuan, Ling Zhao, et al. Regional homogeneity abnormalities in patients with interictal migraine without aura: a resting-state study. *NMR in Biomedicine*. 2012;25(5):806-812.
7. Kai Yuan, Wei Qin, Peng Liu, et al. Reduced fractional anisotropy of corpus callosum modulates inter-hemispheric resting state functional connectivity in migraine patients without aura. 2012;
8. Nouchine Hadjikhani, Noreen Ward, Jasmine Boshyan, et al. The missing link: enhanced functional connectivity between amygdala and visceroceptive cortex in migraine. *Cephalalgia*. 2013;33(15):1264-1268.
9. Chenwang Jin, Kai Yuan, Limei Zhao, et al. Structural and functional abnormalities in migraine patients without aura. *NMR in Biomedicine*. 2013;26(1):58-64.
10. Todd J Schwedt, Bradley L Schlaggar, Soe Mar, et al. Atypical resting-state functional connectivity of affective pain regions in chronic migraine. *Headache: The Journal of Head and Face Pain*. 2013;53(5):737-751.
11. Alessandro Tessitore, Antonio Russo, Alfonso Giordano, et al. Disrupted default mode network connectivity in migraine without aura. *The journal of headache and pain*. 2013;14:1-7.
12. Ting Xue, Kai Yuan, Ping Cheng, et al. Alterations of regional spontaneous neuronal activity and corresponding brain circuit changes during resting state in migraine without aura. *NMR in Biomedicine*. 2013;26(9):1051-1058.
13. Kai Yuan, Ling Zhao, Ping Cheng, et al. Altered structure and resting-state functional connectivity of the basal ganglia in migraine patients without aura. *The journal of pain*. 2013;14(8):836-844.
14. F. H. Maniyar, T. Sprenger, T. Monteith, C. Schankin, P. J. Goadsby. Brain activations in the premonitory phase of nitroglycerin-triggered migraine attacks. *Brain*. Jan 2014;137(Pt 1):232-241. doi:10.1093/brain/awt320
15. Eric A Moulton, Lino Becerra, Adriana Johnson, Rami Burstein, David Borsook. Altered hypothalamic functional connectivity with autonomic circuits and the locus coeruleus in migraine. *PloS one*. 2014;9(4):e95508.

16. Anders Hougaard, FM Amin, S Magon, T Sprenger, E Rostrup, M Ashina. No abnormalities of intrinsic brain connectivity in the interictal phase of migraine with aura. *European Journal of Neurology*. 2015;22(4):702-e746.
17. Kuangshi Li, Yong Zhang, Yanzhe Ning, et al. The effects of acupuncture treatment on the right frontoparietal network in migraine without aura patients. *The Journal of Headache and Pain*. 2015;16:1-10.
18. Faisal Mohammad Amin, Anders Hougaard, Stefano Magon, et al. Change in brain network connectivity during PACAP38-induced migraine attacks: a resting-state functional MRI study. *Neurology*. 2016;86(2):180-187.
19. Gianluca Coppola, Antonio Di Renzo, Emanuele Tinelli, et al. Thalamo-cortical network activity during spontaneous migraine attacks. *Neurology*. 2016;87(20):2154-2160.
20. Duncan J Hodkinson, Rosanna Veggeberg, Aaron Kucyi, et al. Cortico–cortical connections of primary sensory areas and associated symptoms in migraine. *eneuro*. 2016;3(6)
21. David M Niddam, Kuan-Lin Lai, Jong-Ling Fuh, Chih-Ying Naomi Chuang, Wei-Ta Chen, Shuu-Jiun Wang. Reduced functional connectivity between salience and visual networks in migraine with aura. *Cephalalgia*. 2016;36(1):53-66.
22. Laura H Schulte, Arne May. The migraine generator revisited: continuous scanning of the migraine cycle over 30 days and three spontaneous attacks. *Brain*. 2016;139(7):1987-1993.
23. Gioacchino Tedeschi, Antonio Russo, Francesca Conte, et al. Increased interictal visual network connectivity in patients with migraine with aura. *Cephalalgia*. 2016;36(2):139-147.
24. Jilei Zhang, Jingjing Su, Mengxing Wang, et al. Increased default mode network connectivity and increased regional homogeneity in migraineurs without aura. *The journal of headache and pain*. 2016;17:1-9.
25. X Michelle Androulakis, Kaitlin Krebs, B Lee Peterlin, et al. Modulation of intrinsic resting-state fMRI networks in women with chronic migraine. *Neurology*. 2017;89(2):163-169.
26. Nanna Arngrim, Anders Hougaard, Khazar Ahmadi, et al. Heterogenous migraine aura symptoms correlate with visual cortex functional magnetic resonance imaging responses. *Annals of Neurology*. 2017;82(6):925-939.
27. Zhiye Chen, Xiaoyan Chen, Mengqi Liu, Zhao Dong, Lin Ma, Shengyuan Yu. Altered functional connectivity of amygdala underlying the neuromechanism of migraine pathogenesis. *The journal of headache and pain*. 2017;18:1-8.
28. Anders Hougaard, Faisal Mohammad Amin, Henrik BW Larsson, Egill Rostrup, Messoud Ashina. Increased intrinsic brain connectivity between pons and somatosensory cortex during attacks of migraine with aura. *Human brain mapping*. 2017;38(5):2635-2642.
29. Viviana Lo Buono, Lilla Bonanno, Francesco Corallo, et al. Functional connectivity and cognitive impairment in migraine with and without aura. *The journal of headache and pain*. 2017;18:1-6.
30. Laura H Schulte, Angie Allers, Arne May. Hypothalamus as a mediator of chronic migraine: evidence from high-resolution fMRI. *Neurology*. 2017;88(21):2011-2016.
31. Dahua Yu, Kai Yuan, Lin Luo, et al. Abnormal functional integration across core brain networks in migraine without aura. *Molecular Pain*. 2017;13:1744806917737461.

32. Jilei Zhang, Jingjing Su, Mengxing Wang, et al. The sensorimotor network dysfunction in migraineurs without aura: a resting-state fMRI study. *Journal of neurology*. 2017;264:654-663.
33. Faisal Mohammad Amin, Anders Hougaard, Stefano Magon, et al. Altered thalamic connectivity during spontaneous attacks of migraine without aura: a resting-state fMRI study. *Cephalalgia*. 2018;38(7):1237-1244.
34. Noemi Meylakh, Kasia K Marciszewski, Flavia Di Pietro, Vaughan G Macefield, Paul M Macey, Luke A Henderson. Deep in the brain: changes in subcortical function immediately preceding a migraine attack. *Human brain mapping*. 2018;39(6):2651-2663.
35. G. Coppola, A. Di Renzo, B. Petolicchio, et al. Aberrant interactions of cortical networks in chronic migraine: A resting-state fMRI study. *Neurology*. May 28 2019;92(22):e2550-e2558. doi:10.1212/wnl.0000000000007577
36. Mi Ji Lee, Bo-yong Park, Soohyun Cho, Sung Tae Kim, Hyunjin Park, Chin-Sang Chung. Increased connectivity of pain matrix in chronic migraine: a resting-state functional MRI study. *The journal of headache and pain*. 2019;20:1-10.
37. Antonio Russo, Alessandro Tessitore, Marcello Silvestro, et al. Advanced visual network and cerebellar hyperresponsiveness to trigeminal nociception in migraine with aura. *The Journal of Headache and Pain*. 2019;20:1-10.
38. Gianluca Coppola, Antonio Di Renzo, Barbara Petolicchio, et al. Increased neural connectivity between the hypothalamus and cortical resting-state functional networks in chronic migraine. *Journal of neurology*. 2020;267:185-191.
39. Nazia Karsan, Pyari R Bose, Owen O'Daly, Fernando O Zelaya, Peter J Goadsby. Alterations in functional connectivity during different phases of the triggered migraine attack. *Headache: The Journal of Head and Face Pain*. 2020;60(7):1244-1258.
40. Laura H Schulte, Mareike M Menz, Jan Haaker, Arne May. The migraineur's brain networks: Continuous resting state fMRI over 30 days. *Cephalalgia*. 2020;40(14):1614-1621.
41. Dániel Veréb, Nikoletta Szabó, Bernadett Tuka, et al. Temporal instability of salience network activity in migraine with aura. *Pain*. 2020;161(4):856-864.
42. N. Maleki, E. Szabo, L. Becerra, et al. Ictal and interictal brain activation in episodic migraine: Neural basis for extent of allodynia. *PLoS One*. 2021;16(1):e0244320. doi:10.1371/journal.pone.0244320
43. Daniele Martinelli, Gloria Castellazzi, Roberto De Icco, et al. Thalamocortical connectivity in experimentally-induced migraine attacks: A pilot study. *Brain Sciences*. 2021;11(2):165.
44. Willebrordus PJ van Oosterhout, Anne M van Opstal, Guus G Schoonman, et al. Hypothalamic functional MRI activity in the initiation phase of spontaneous and glyceryl trinitrate-induced migraine attacks. *European Journal of Neuroscience*. 2021;54(3):5189-5202.
45. Zhijian Cao, Wenjing Yu, Zhengxiang Zhang, et al. Decreased Gray Matter Volume in the Frontal Cortex of Migraine Patients with Associated Functional Connectivity Alterations: A VBM and rs-FC Study. *Pain Research and Management*. 2022;2022(1):2115956.
46. C. Gollion, F. Lerebours, F. Nemmi, et al. Insular functional connectivity in migraine with aura. *J Headache Pain*. Aug 19 2022;23(1):106. doi:10.1186/s10194-022-01473-1
47. Camillo Porcaro, Antonio Di Renzo, Emanuele Tinelli, et al. A hypothalamic mechanism regulates the duration of a migraine attack: insights from microstructural and temporal complexity of cortical functional networks analysis. *International Journal of Molecular Sciences*. 2022;23(21):13238.

48. Junseok Jang, Sungyeong Ryu, Dong Ah Lee, Kang Min Park. Are there network differences between the ipsilateral and contralateral hemispheres of pain in patients with episodic migraine without aura? *Annals of Clinical Neurophysiology*. 2023;25(2):93-102.
49. P. Y. Henry, J. Vernhiet, J. M. Orgogozo, J. M. Caille. Cerebral blood flow in migraine and cluster headache. Compartmental analysis and reactivity to anaesthetic depression. *Res Clin Stud Headache*. 1978;6:81-88.
50. Fumihiko Sakai, John Stirling Meyer. Regional cerebral hemodynamics during migraine and cluster headaches measured by the 133Xe inhalation method. *Headache: The Journal of Head and Face Pain*. 1978;18(3):122-132.
51. Annette Æbelholt Krabbe, Leif Henriksen, Jes Olesen. Tomographic determination of cerebral blood flow during attacks of cluster headache. *Cephalalgia*. 1984;4(1):17-23.
52. Jen-Chuen Hsieh, Jan Hannerz, Martin Ingvar. Right-lateralised central processing for pain of nitroglycer-induced cluster headache. *Pain*. 1996;67(1):59-68.
53. Vittorio Di Piero, Fabrizio Fiacco, David Tombari, Patrizia Pantano. Tonic pain: a SPET study in normal subjects and cluster headache patients. *Pain*. 1997;70(2-3):185-191.
54. A. May, A. Bahra, C. Büchel, R. S. Frackowiak, P. J. Goadsby. Hypothalamic activation in cluster headache attacks. *Lancet*. Jul 25 1998;352(9124):275-278. doi:10.1016/s0140-6736(98)02470-2
55. A May, J Ashburner, C Büchel, et al. Correlation between structural and functional changes in brain in an idiopathic headache syndrome. *Nature medicine*. 1999;5(7):836-838.
56. A May, A Bahra, C Buchel, RSJ Frackowiak, PJ Goadsby. PET and MRA findings in cluster headache and MRA in experimental pain. *Neurology*. 2000;55(9):1328-1335.
57. T Sprenger, H Boecker, TR Tolle, G Bussone, A May, M Leone. Specific hypothalamic activation during a spontaneous cluster headache attack. *Neurology*. 2004;62(3):516-517.
58. Raffaele Lodi, Giulia Pierangeli, Caterina Tonon, et al. Study of hypothalamic metabolism in cluster headache by proton MR spectroscopy. *Neurology*. 2006;66(8):1264-1266.
59. SJ Wang, JF Lirng, JL Fuh, JJ Chen. Reduction in hypothalamic 1H-MRS metabolite ratios in patients with cluster headache. *Journal of Neurology, Neurosurgery & Psychiatry*. 2006;77(5):622-625.
60. T Sprenger, KV Ruether, H Boecker, et al. Altered metabolism in frontal brain circuits in cluster headache. *Cephalalgia*. 2007;27(9):1033-1042.
61. Nicola Morelli, Ilaria Pesaresi, Gianfranco Cafforio, et al. Functional magnetic resonance imaging in episodic cluster headache. *The journal of headache and pain*. 2009;10:11-14.
62. Maria A Rocca, Paola Valsasina, Martina Absinta, et al. Central nervous system dysregulation extends beyond the pain-matrix network in cluster headache. *Cephalalgia*. 2010;30(11):1383-1391.
63. En-chao Qiu, Sheng-yuan Yu, Ruo-zhuo Liu, Yan Wang, Lin Ma, Li-xia Tian. Altered regional homogeneity in spontaneous cluster headache attacks: a resting-state functional magnetic resonance imaging study. *Chinese Medical Journal*. 2012;125(4):705-709.
64. A Steinberg, R Axelsson, L Ideström, S Müller, AIM Nilsson Remahl. White blood cell SPECT during active period of cluster headache and in remission. *European Journal of Neurology*. 2012;19(2):220-225.

65. Nicola Morelli, Eugenia Rota, Sara Gori, et al. Brainstem activation in cluster headache: an adaptive behavioural response? *Cephalalgia*. 2013;33(6):416-420.
66. Kun-Hsien Chou, Fu-Chi Yang, Jong-Ling Fuh, et al. Altered white matter microstructural connectivity in cluster headaches: a longitudinal diffusion tensor imaging study. *Cephalalgia*. 2014;34(13):1040-1052.
67. Enchao Qiu, Lixia Tian, Yan Wang, Lin Ma, Shengyuan Yu. Abnormal coactivation of the hypothalamus and salience network in patients with cluster headache. *Neurology*. 2015;84(14):1402-1408.
68. Fu-Chi Yang, Kun-Hsien Chou, Jong-Ling Fuh, et al. Altered hypothalamic functional connectivity in cluster headache: a longitudinal resting-state functional MRI study. *Journal of Neurology, Neurosurgery & Psychiatry*. 2015;86(4):437-445.
69. Enrico B Arkink, Nicole Schmitz, Guus G Schoonman, et al. The anterior hypothalamus in cluster headache. *Cephalalgia*. 2017;37(11):1039-1050.
70. Kun-Hsien Chou, Fu-Chi Yang, Jong-Ling Fuh, et al. Bout-associated intrinsic functional network changes in cluster headache: a longitudinal resting-state functional MRI study. *Cephalalgia*. 2017;37(12):1152-1163.
71. Péter Faragó, Nikoletta Szabó, Eszter Tóth, et al. Ipsilateral alteration of resting state activity suggests that cortical dysfunction contributes to the pathogenesis of cluster headache. *Brain topography*. 2017;30:281-289.
72. Stefania Ferraro, Anna Nigri, Maria Grazia Bruzzone, et al. Defective functional connectivity between posterior hypothalamus and regions of the diencephalic-mesencephalic junction in chronic cluster headache. *Cephalalgia*. 2018;38(13):1910-1918.
73. Sam Yeol Ha, Kang Min Park. Alterations of structural connectivity in episodic cluster headache: a graph theoretical analysis. *Journal of Clinical Neuroscience*. 2019;62:60-65.
74. Catherine D Chong, Maria Aguilar, Todd J Schwedt. Altered hypothalamic region covariance in migraine and cluster headache: a structural MRI study. *Headache: The Journal of Head and Face Pain*. 2020;60(3):553-563.
75. Antonio Giorgio, Chiara Lupi, Jian Zhang, et al. Changes in grey matter volume and functional connectivity in cluster headache versus migraine. *Brain imaging and behavior*. 2020;14:496-504.
76. Maike Möller, Jan Mehnert, Arne May. Hypothalamic activation discriminates painful and non-painful initiation of the trigeminal autonomic reflex–an fMRI study. *Cephalalgia*. 2020;40(1):79-87.
77. Stefania Ferraro, Jean Paul Medina, Anna Nigri, et al. Mesocorticolimbic system abnormalities in chronic cluster headache patients: A neural signature? *Cephalalgia*. 2022;42(10):1039-1049.
78. Roberta Messina, Maria A Rocca, Paola Valsasina, Paolo Misci, Massimo Filippi. Clinical correlates of hypothalamic functional changes in migraine patients. *Cephalalgia*. 2022;42(4-5):279-290.
